# Effects of antibiotic resistance genes on health risks of rivers in habitat of wild animals under human disturbance – based on analysis of antibiotic resistance genes and virulence factors in microbes of river sediments

**DOI:** 10.1002/ece3.11435

**Published:** 2024-05-24

**Authors:** Rongpan Hu, Minxing Ren, Sumei Liang, Shuzhen Zou, Dayong Li

**Affiliations:** ^1^ Key Laboratory of Southwest China Wildlife Resources Conservation of Ministry of Education China West Normal University Nanchong China; ^2^ Key Laboratory of Conservation Biology of Rhinopithecus Roxellana at China West Normal University of Sichuan Province China West Normal University Nanchong China; ^3^ Liziping Giant Panda's Ecology and Conservation Observation and Research Station of Sichuan Province Science and Technology Department of Sichuan Province Chengdu China

**Keywords:** environmental health assessment, microbial risk, nature reserve, pathogenic antibiotic‐resistant bacteria

## Abstract

Studying the ecological risk of antibiotic resistance genes (ARGs) to wild animals from human disturbance (HD) is an important aspect of “One Health”. The highest risk level of ARGs is reflected in pathogenic antibiotic‐resistant bacteria (PARBs). Metagenomics was used to analyze the characteristics of PARBs in river sediments. Then, the total contribution of ARGs and virulence factors (VFs) were assessed to determine the health risk of PARBs to the rivers. Results showed that HD increased the diversity and total relative abundance of ARG groups, as well as increased the kinds of PARBs, their total relative abundance, and their gene numbers of ARGs and VFs. The total health risks of PARBs in wild habitat group (CK group), agriculture group (WA group), grazing group (WG group), and domestic sewage group (WS group) were 0.067 × 10^−3^, −1.55 × 10^−3^, 87.93 × 10^−3^, and 153.53 × 10^−3^, respectively. Grazing and domestic sewage increased the health risk of PARBs. However, agriculture did not increase the total health risk of the rivers, but agriculture also introduced new pathogenic mechanisms and increased the range of drug resistance. More serious was the increased transfer risk of ARGs in the PARBs from the rivers to wild animals under agriculture and grazing. If the ARGs in the PARBs are transferred from the rivers under HD to wild animals, then wild animals may face severe challenges of acquiring new pathogenic mechanisms and developing resistance to antibiotics. Further analysis showed that the total phosphorus (TP) and dissolved organic nitrogen (DON) were related to the risk of ARGs. Therefore, controlling human emissions of TP and DON could reduce the health risk of rivers.

## Graphical abstract

Studying the ecological risk of antibiotic resistance genes (ARGs) to wild animals from human disturbance (HD) is an important aspect of “One Health”. The highest risk level of ARGs is reflected in pathogenic antibiotic‐resistant bacteria (PARBs). We tested the effect of HD on the health risk of PARBs in rivers in the habitat of wild animals.

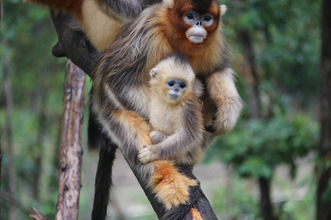

## 1 INTRODUCTION

“One Health” suggests that model of human–animal–environmental health is closely related, but in fact, the model is almost controlled by humans (Zinsstag et al., 2023). The harmonious balance of the model could be disrupted by negative human disturbance (HD), resulting in the degradation of natural environment (Acheampong & Opoku, 2023; Jokanović et al., 2021; Zhang et al., 2020) and the deterioration of animal health (Doherty et al., 2021; Miller et al., 2023). Nature reserves of wild animals play a pivotal role in protecting rare and endangered wild animals and ensuring ecological security (Wang, Liu, et al., 2021). In China, to harmonize the protection of wild animals and the development of human society, numerous policies have been introduced in recent decades, but the contradictions between the two are often difficult to reconcile (Guo & Wu, 2023), and the ecological networks in nature reserves face escalating challenges as a result of increasing HD (Gu et al., 2023). The presence of significant HD in the nature reserves of wild animals, as mentioned, establishes a certain antagonistic relationship with the habitat of wild animals (Qiu, 2015). Thus, studying the ecological risks of HD in nature reserves of wild animals becomes crucial for understanding and mitigating the challenges of the contradictions between the protection of wild animals and the development of human society.

Microbial risk is indeed a significant aspect of “One Health” (Wu et al., 2023), and it is also an important component for assessing environmental health risks (Abdugheni et al., 2023). Antibiotic resistance genes (ARGs) in microbes represent a critical concern when evaluating microbial risks (Li et al., 2022; Shao et al., 2018). HD plays a crucial role in fostering the proliferation of ARGs (Czekalski et al., 2015; Pruden et al., 2012). Aquatic ecosystems, frequently impacted by humans, serve as reservoirs for ARGs and provide an optimal environment for the accumulation and dissemination of ARGs (Marti et al., 2014; Rout et al., 2024; Rout, Das, et al., 2023). And rivers are one of the main containers for livestock and poultry farms runoff, fertilizer runoff and rural domestic sewage, which are widely recognized as significant contributors to the accumulation of ARGs in rivers (Chen et al., 2015; Cheng et al., 2013; Mware et al., 2022).

In China, rivers crisscross the nature reserves of wild animals, which are suffering from HD such as farming, animal husbandry, and domestic sewage emissions that are sources of ARGs (Lu et al., 2022; Zhang et al., 2014). For the ARGs in rivers could enter the body by drinking water (Zhang, Qin, et al., 2021), it is indicated that the ARGs in the rivers with nature reserves of wild animals could be moved to wild animals by drinking. The gut microbiota plays a vital role in the health of wild animals by providing essential nutritional services and protection against the invasion of intestinal pathogens (Fackelmann et al., 2021), and regulating multiple aspects of microbial metabolite pools that could affect the gut barrier and the polarization of immune cells (Tan et al., 2023). Many studies have shown that ARGs can be transferred into the gut microbiota (Khan et al., 2020; Lamberte & van Schaik, 2022; Rolain, 2013), if the ARGs entry into the gut will have a significant impact on the gut microbiota, which may lead to various diseases and represent unknown risks to wild animals (Zhu et al., 2021).

However, not all ARGs pose risks to ecosystems. Most studies have found that ARGs are present even in places where humans have never been (Kim et al., 2022; Luo et al., 2021; Zeng et al., 2019). Therefore, when assessing the risk of ARGs to ecosystems, the risk level of ARGs should be categorized, with pathogens containing both ARGs and virulence factors (VFs) representing the highest risk level of ARGs (Martínez et al., 2015). On the one hand, drug‐resistant pathogens cause disease and render antibiotic treatment ineffective (Pan et al., 2020); on the other hand, co‐adaptation of virulence and resistance often gives the pathogens a greater advantage, which may pose unknown ecological risks. In metagenomic assembly genomes (MAGs) of microbes, if a MAG contains one or more ARGs and VFs, the MAG could be potential pathogenic antibiotic‐resistant bacterium (PARB) (Liang et al., 2020).

Some studies have used PARBs to assess microbial risks to ecosystems under HD. For example, Liang et al. (2020) studied the occurrence of PARBs in aquatic environments impacted by HD and found that HD leads to high microbial risk, which was reflected in the high proportion of MAGs identified as PARBs, as well as the high abundance and density of PARBs in human‐disturbed areas, also confirmed that 81.8% of PARBs are related to known pathogenic taxa. Zou, Xiao, et al. (2023) investigated the effect of wastewater treatment plants effluent discharge on the microbiological risks of low‐flow rivers and also found the high proportion of PARBs in effluent‐dominated rivers. Both of these studies indicated that intragenomic ARGs‐VFs coexistence seemed more likely to occur in regions affected by HD, also indicated the higher microbial risks in rivers under HD. Therefore, the occurrence of PARBs in river ecosystems should be of great concern. However, there is still a lack of research on the use of PARBs to analyze the health risks of ARGs to rivers in nature reserves of wild animals, where there is often a close interaction between humans and wild animals.

Studying the factors that influence the risk of ARGs in rivers under HD plays an important role in reducing the ecological risk of HD to rivers (Na et al., 2018; Rout, Tripathy, et al., 2023). HD will not only directly increase the risk of ARGs (Zhang et al., 2022), but also increase the risk of ARGs by altering environmental factors, which have been proposed for shaping ARG profiles (Zhao et al., 2023). In addition, the environmental factors of rivers have the potential to induce shifts in the structure of river microflora (Wang, Fan, et al., 2021), which is also closely related to the risk of ARGs (Zhou et al., 2017). Furthermore, the spread of ARGs in pathogens from rivers in habitats of wild animals is a matter of great concern (Zhang, Gaston, et al., 2021). ARGs could be horizontally transferred by mobile genetic elements (MGEs) in the environment (Jeon et al., 2023), thus the MGEs in PARBs may play an important role in the transfer of ARGs from rivers to the gut microbiome of wild animals. Now in China, the case of HD for survival is unavoidable, and it is crucial to study the causes of the factors affecting the risk of ARGs from the rivers to wild animals in the habitats under HD.

Our study aims to explore the effects of HD of agriculture, grazing, and domestic sewage in China's nature reserves of wild animals on the health risks of ARGs in rivers, as well as to find the main factors affecting the health risks of ARGs from rivers to wild animals. The characteristics of ARGs in microbes of river sediments were examined to study the impact of HD on the ARGs, the total contribution of ARGs and VFs in PARBs were assessed by factor analysis to measure the risk to river health, and the plasmids that carried ARGs in PARBs were identified to determine the transfer risk of ARGs in PARBs. Lastly, the influence of environmental factors of the river sediments on health risks of ARGs to the rivers were explored. Our study could be offered informed perspectives for sustainable human activities in the nature reserves of wild animals, aiming at a “win‐win” scenario that balances the economic and life development of the inhabitants of these reserves with the imperative of wildlife conservation.

## 2 MATERIALS AND METHODS

### 2.1 Sample collection

Baihe National Nature Reserve (104°01′‐104°12′ E, 33°10′‐33°22′ N) is a habitat for endangered wild animals such as giant pandas (*Ailuropoda melanoleuca*) and snub‐nosed golden monkeys (*Rhinopithecus roxellana*) in China. Our previous study found that HD increased the health risk of gut microbes in golden monkeys because primates are more likely to acquire ARGs and VFs from sources of HD (Zou, Yuan, et al., 2023). The investigation showed that HD such as grazing, farmland cultivation, and domestic sewage discharge were typical and severe in the Baihe National Nature Reserve. Therefore, we chose to conduct our study in the Baihe National Nature Reserve.

**FIGURE 1:**
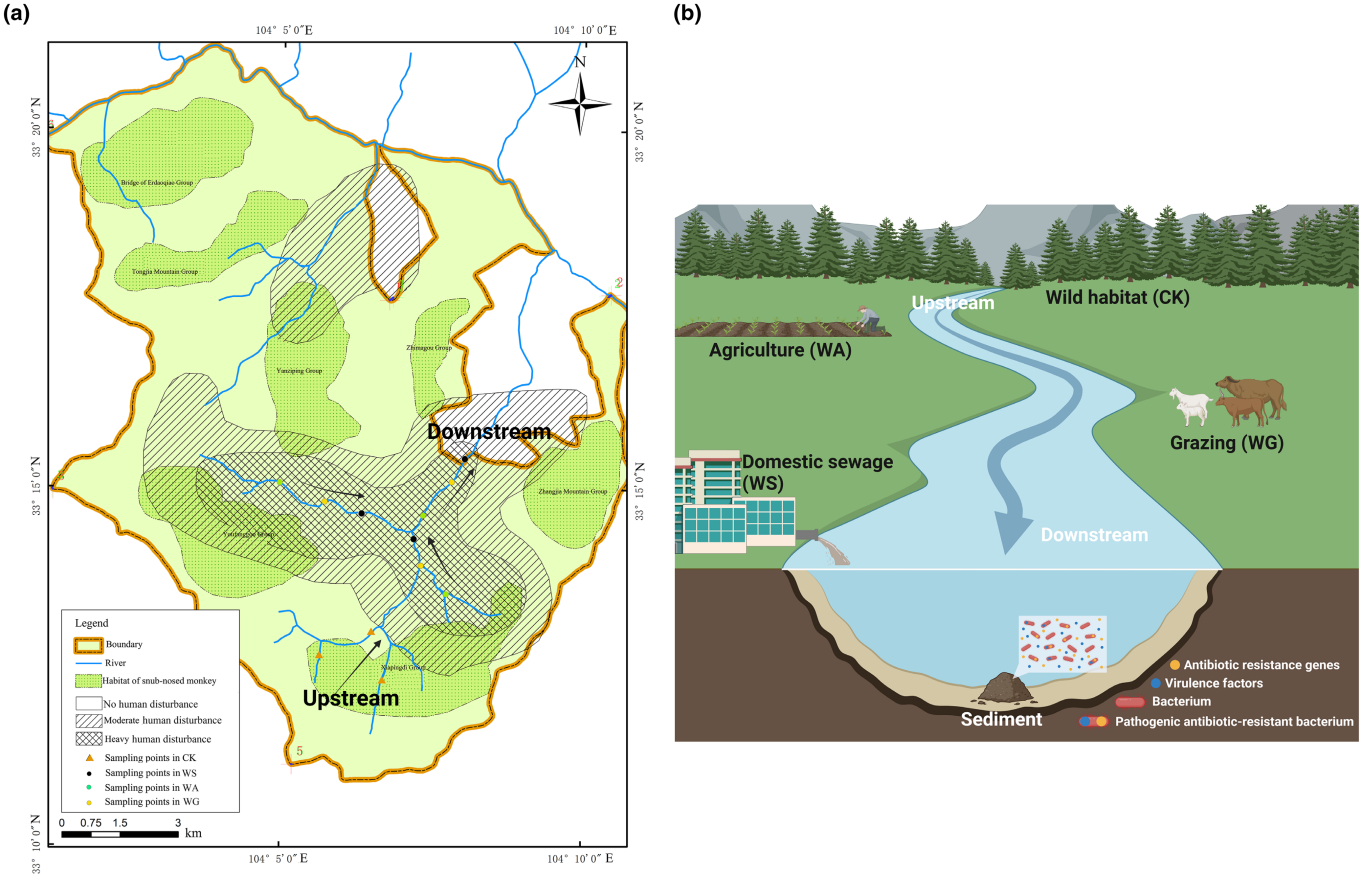
(a) Baihe National Nature Reserve and sampling points. (b) Overview of sampling surroundings. Image created with BioRender.com, with permission.

In the Baihe National Nature Reserve, the river flows from southwest to northeast. We sampled river sediments from the areas disturbed by human activities, including agriculture, grazing, and domestic sewage; the sediments of the rivers under agriculture, grazing, and domestic sewage were defined as WA group, WG group, and WS group, respectively. At the same time, we sampled sediments from the upstream of the rivers that had never been disturbed by humans (wild habitat) and defined these sediment samples as check control (CK group). Although some sections of the downstream of the rivers are undisturbed by humans, we could not be sure whether pollutants from HD had migrated downstream (north) with the flow of the water in the rivers and affected the sediments in the wild habitat. In addition, there are roads built along the northern part of the rivers where wild animals do not occur. Sampling was conducted in June 2022, with samples collected from three different locations near the center of rivers at each site. The column sampler was used to collect the top 10 centimeters of sediments. To minimize the impact of geographic patterns, the distance among our sampling sites cannot be too far. In order to minimize the errors due to sampling, we set up three replicates of each type of HD. Twelve sediment samples in total were then carefully preserved in dry ice and transported to the laboratory (Figure 1).

### 2.2 DNA extraction

DNA extraction from the sediments was conducted using the FastDNA® Spin Kit for Soil (MP Biomedicals, France). The quantification and purity of the extracted DNA were subsequently assessed using a NanoDrop™ spectrophotometer. DNA samples meeting the criteria of an absorbance ratio between 260 and 280 nm falling in the range of 1.8–2.0 were considered suitable for metagenomic sequencing. The process of DNA extraction was conducted by the laboratory at Shanghai Magi Biological Company, China (Meiji Biological Medicine Co., Ltd., Shanghai, China). Each sample was extracted three times as described above, and eligible DNA from all three times was pooled for subsequent analysis.

### 2.3 Metagenomic sequencing

We used Covaris M220 (Gene Company Limited, Shanghai, China) to fragment the obtained DNA and subsequently screened fragments of approximately 400 bp to construct the paired‐end library using NEXTFLEX Rapid DNA‐Seq according to the manufacturer's instructions. HiSeq 2000 (Illumina Inc., San Diego, CA, USA) was selected for metagenomic sequencing of the paired‐end library at Majorbio Bio‐Pharm Technology Co., Ltd. (Shanghai, China). The data were deposited into the NCBI Sequence Read Archive (SRA) database under accession number PRJNA1044275. For the obtained metagenomic data, we used fastp to perform quality clipping of adapter sequences from reads and remove low‐quality reads (reads <50 bp in length with an average mass value of <20 and containing N bases) (Zhang et al., 2017). The reads were compared with the host genome sequence using BWA software (https://bio‐bwa.sourceforge.net). Reads with high similarity were removed to obtain clean reads for subsequent analysis. Next, clean reads were assembled using Megahit for contigs, and contigs with the shortest sequence longer than 300 bp were retained (Li et al., 2015). We used MetaGene to perform an open reading frame prediction of the retained contigs (length of reads ≥100 bp), and the per‐base coverage depth across all contigs was calculated by mapping raw reads from each sample (Zhu et al., 2010). CD‐HIT was used to construct the coverage >90% and identity >90% of predicted gene sequences of all samples as a nonredundant gene contig. The identity ≥0.95 of nonredundant gene contig was blasted using SOAPaligner, and gene abundance of the nonredundant gene contigs was subsequently calculated. Then, the nonredundant gene contig was predicted as the open reading frame (ORF), and the ORFs were used in card_v3.0.9 (the Comprehensive Antibiotic Research Database) to obtain ARGs (http://arpcard.mcmaster.ca, (accessed on 28 November 2022); blastp, e‐value: 1 × 10^−5^). And plasmids in ACLAM were used to obtain MGEs (blastp, e‐value: 1 × 10^−5^). An identity >80% of ARGs and MGEs were selected for analysis.

To compare coverage between different samples, the coverage of ARG‐like ORFs and MGE‐like ORFs was normalized using the data size of each sample (copies/Gb) (Ma et al., 2016).

### 2.4 Assembly of MAGs

The filtered clean reads were grouped by sampling into four groups (CK group, WA group, WG group and WS group) and co‐assembled using MEGAHIT v1.13 with default parameters (Li et al., 2015). These co‐assembled contigs were clustered to recover metagenomes using metaBAT (contig length ≥1000 bp) (Kang et al., 2019). The assembled bacterial genomes were further refined to produce high‐quality individual genomes using the built‐in refining module of MetaWRAP (Uritskiy et al., 2018), with the selection criteria of >50% completeness and <10% contamination. Following that procedure, all metagenome data were refined to remove redundant assemblages and then annotated for taxonomic classifications using the Genome Taxonomy Database (GTDB; v1.4.0) (Chaumeil et al., 2019). Next, high‐quality bins were reassembled to reconstruct the MAGs using SPAdes (Bankevich et al., 2012). The assembled genomes of MAGs were used to blast the database of ARGs (blastp, evalue: 1 × 10^−5^, identity >70%, http://arpcard.mcmaster.ca) and the database of VFs (blastp, evalue: 1 × 10^−5^, identity >70%, http://www.mgc.ac.cn/VFs/) to obtain PARBs (Wu et al., 2022).

The coverage of each MAG was calculated as the average scaffold coverage, and each scaffold was weighed by its length in base pairs. Then, the relative abundance of each MAG was calculated as its coverage divided by the total coverage of all genome bins.

Thus, the relative abundance of each MAG was calculated as the number of reads (based on average coverage) aligning to the MAG normalized by the total number of reads in the sample. The calculation formula was as follows:

(1) $$A_{i}=C_{i}\times \left(N_{i}/N\right)$$

where *A*_*i*_, *C*_*i*_, and *N*_*i*_ represent the relative abundances, coverage, and contig numbers of *i* MAG, respectively. *N* is the total number of reads in the samples.

### 2.5 Identification of plasmids

To further evaluate the risk of transmission, it is imperative to ascertain the location of ARGs in each PARB. To accomplish this, we used the plasmid prediction tool RFPlasmid to identify the plasmids in MAGs (van der Graaf‐van Bloois et al., 2021). The MAG was uploaded to the webpage (klif.uu.nl/rfplasmid/), where the Generic model was selected, and the submission was made. Then, we selected the scaffolds that identified as plasmids (votes plasmid >0.5), and the ARGs identified in scaffolds of MAGs were compared with the plasmid scaffolds to analyze the ARGs carried by the plasmid in PARBs.

### 2.6 River health risk assessment

**FIGURE 2:**
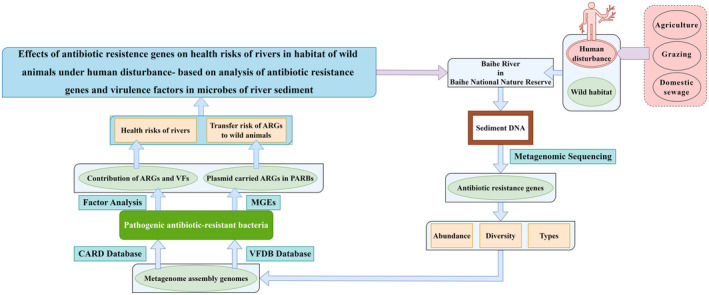
Conceptual framework of the health risk assessment.

Four independent variables were considered, including the types and numbers of ARGs and VFs in each PARB in the CK group, the WA group, the WG group and the WS group. To quantify these variables, we tabulated the classes and gene counts of ARGs and VFs in each PARB for the four groups. Subsequently, we conducted factor analysis to extract the principal component features based on these variables. The total scores of the principal component features were computed using Formula (2). The higher scores indicated the greater contribution of ARGs and VFs to the risk of each PARB. Since the health risk of a PARB is influenced by its abundance, we determined the health risk for each PARB using Formula (3). Finally, the total health risk for all PARBs in each group was calculated using Formula (4). The conceptual framework of the health risk assessment for rivers and wild animals is shown in Figure 2.

(2) $$F_{i}=\sum_{1}^{i}S_{i}\times W_{i}$$

(3) $${\mathrm{HR}}_{i}=F_{i}\times A_{i}$$

(4) $$\mathrm{THR}=\sum_{1}^{n}{\mathrm{HR}}_{i}$$

where *i* is the number of component features considered in each of the four groups, *S*_*i*_ is the score of the component features, *W*_*i*_ is the weight of *S*_*i*_ and *F*_*i*_ is the total score of the PARB. *HR*_*i*_ is the health risk of one individual PARB, and *THR* is the total health risk of PARBs in each group. The higher THR value indicates a greater health risk to the river ecosystem.

### 2.7 Environmental factor measurements

We used the pH meter (PHS‐3C, China) to measure the pH of the sediments. Available phosphorus (AP) was determined using the sodium bicarbonate extraction‐molybdenum‐antimony anti‐spectrophotometric method (Olsen, 1954). Available potassium (AK) extracted using the 1 M CH_3_COONH_4_ solution and then analyzed using the flame atomic absorption spectrophotometer (Varian AA240, USA). Slowly available potassium (SAK) extracted using the COD Digestion instrument (Hach DRB200, China), and flame photometer (Perkin Pin AAcle 900F, USA) was used for measurement. Dissolved organic carbon (DOC) was extracted using MK_2_SO_4_ solution and quantified using a TOC‐TN analyzer (Shimadzu, Japan) (Shi et al., 2018). The content of dissolved organic nitrogen (DON) was determined by calculating the difference between total nitrogen (TN) and inorganic nitrogen, and TN was determined using the Kjeldahl method. Total phosphorus (TP) was determined using the sodium hydroxide melting‐molybdenum antimony anti‐colorimetric method. Total potassium (TK) was determined using the sodium hydroxide melting‐flame atomic absorption spectrophotometry method.

### 2.8 Statistical analysis and visualization

In our analysis, one‐way analysis of variance (ANOVA) and *t*‐tests were used to examine the differences between samples for ARGs with a significance level of *p* < .05 using SPSS Statistics 27 (IBM, Armonk, USA). The contig numbers of ARGs and VFs at the type level and microbes were used to calculate their Simpson's index using the R package “vegan” (Oksanen et al., 2017). Principal component analysis (PCA, Canoco 5.0, Microcomputer Power, New York, NY, USA) was used to investigate the relationship between environmental factors and microbial characteristics. Histograms and heatmaps, and Pearson's correlation analysis were performed using OriginPro 2021. Map was created using ArcGIS 10.2 (ESRI Corp. 2013).

## 3 RESULTS

### 3.1 The effect of HD on the ARGs of river sediment microbes in the reserve of wild animals

**FIGURE 3:**
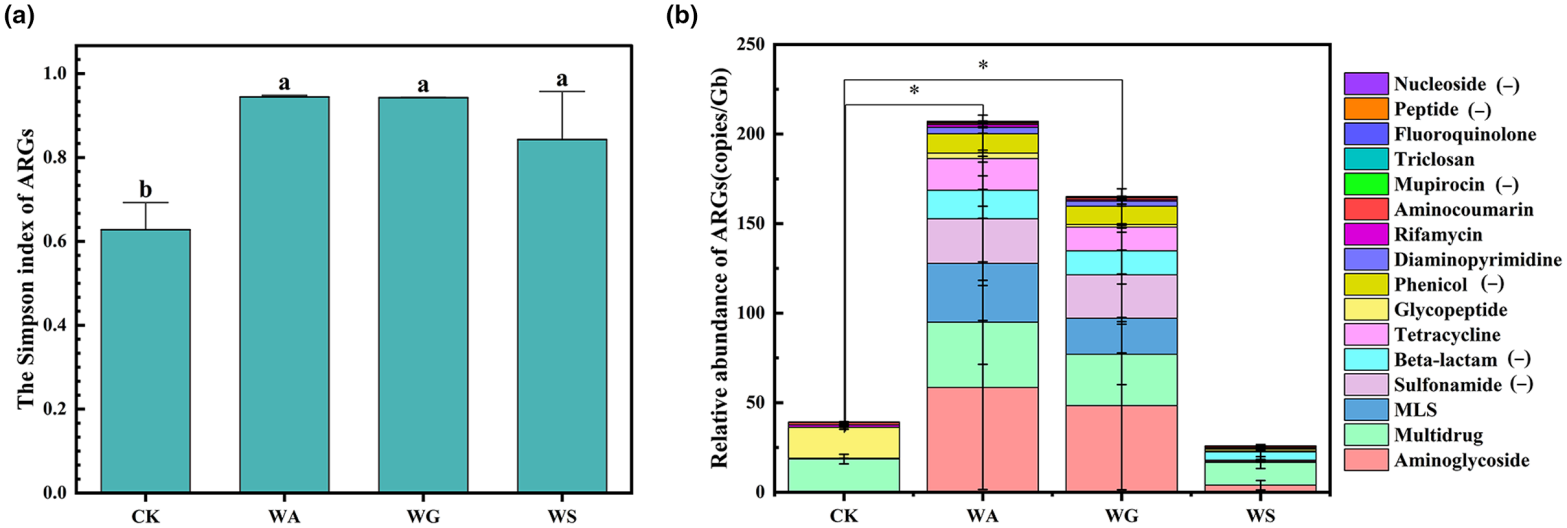
The effect of HD on ARGs of river sediment microbes in the reserve of wild animals. (a) The Simpson's index of ARGs. Different letters indicate significant differences in the Simpson's index between the various groups (*p* < .05). (b) The relative abundance and composition of ARGs. The “*” indicates a significant difference in total relative abundance between the two groups (*p* < .05). The symbol “(−)” denotes the absence of specific classes of ARGs in the CK group.

The Simpson's index of ARGs in river sediments of the WA group, WG group, and WS group were significantly higher than that of the CK group (Figure 3a; ANOVA, *p* < .05). The total relative abundance of ARGs in the WA group and WG group were significantly higher than that in the CK group (Figure 3b; *t*‐test, *p* < .05). A total of 16 classes of ARGs were identified in river sediment samples from four groups, of which Nucleoside, Peptide, Mupirocin, Phenicol, Beta‐lactam, and Sulfonamide were exclusively found in sediments impacted by HD but not detected in the CK group (Figure 3b).

### 3.2 The effect of HD on PARBs of river sediment microbes in the reserve of wild animals

**FIGURE 4:**
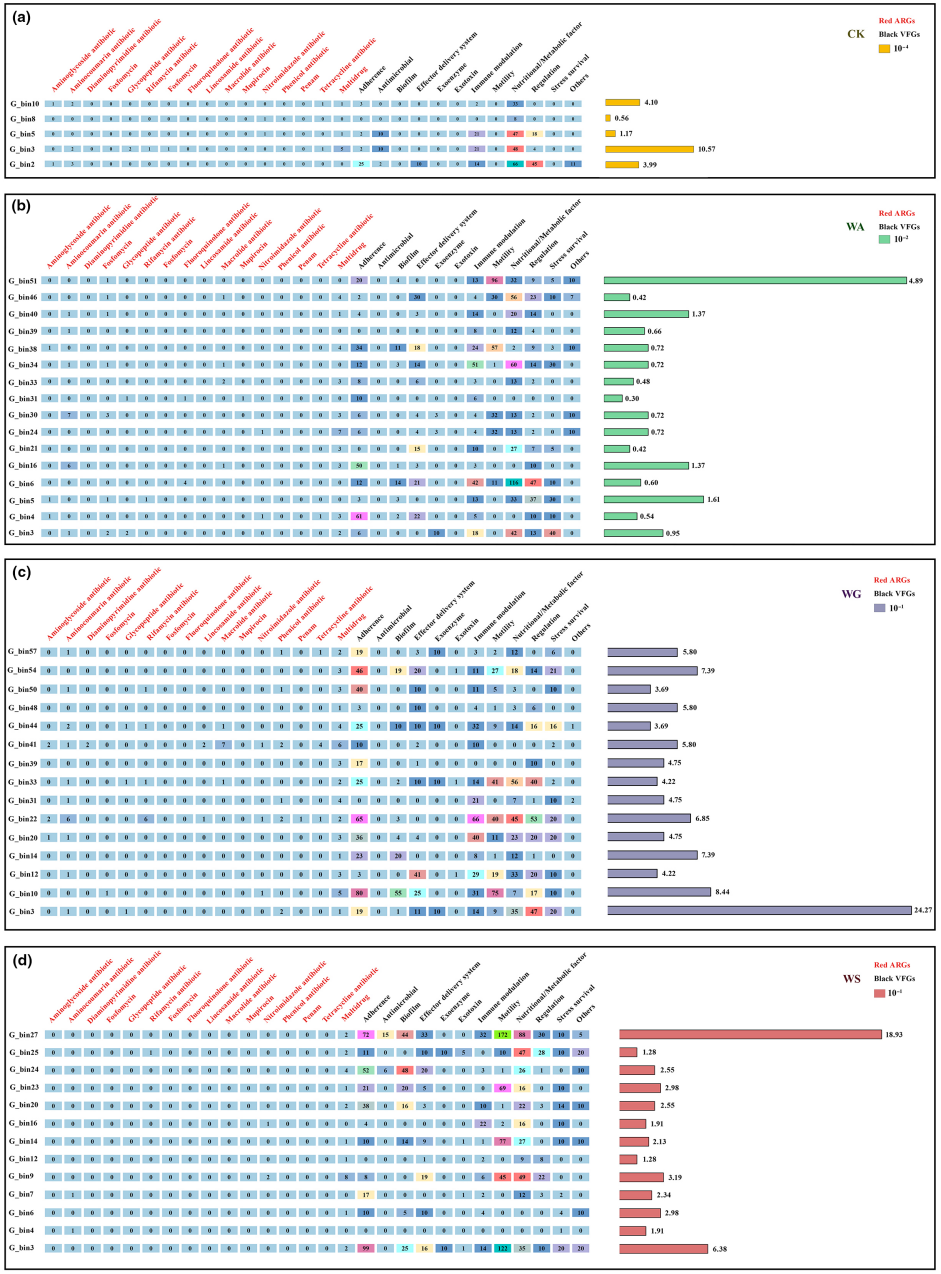
The effect of HD on the relative abundance of potential PARBs and their ARGs and VFs numbers in each group (a‐d).

In total, there were 157 kinds of MAGs in the river sediments of nature reserve of wild animals. While only 49 kinds of MAGs were identified as potential PARBs (Table S1). Among these MAGs, there were five kinds of PARBs in the CK group, 16 kinds of PARBs in the WA group, 15 kinds of PARBs in the WG group, and 13 kinds of PARBs in the WS group. The characteristics of each PARB are shown in Figure 4. All ARGs in the PARBs were collectively categorized into 16 different classes. Among these classes, multidrug was the most prevalent class of ARGs, with 38 kinds out of 49 kinds of PARBs carried it. The ARGs of Diaminopyrimidine, Fosfomycin, Fluoroquinolone, Lincosamide, Macrolide, Mupirocin, Phenicol, and Penam were exclusively present in PARBs affected by HD, while they were absent in the CK group. In addition, VFs were classified into 12 distinct classes in all PARBs. Biofilm, Exoenzyme, Exotoxin, Motility, and Stress survival were only found in PARBs affected by HD but not detected in the CK group. The total relative abundance of PARBs in the CK group, WA group, WG group, and WS group were 0.002, 0.165, 10.182, and 5.042, respectively (Figure S1).

### 3.3 The effect of HD on the health risk of the river in the reserve of wild animals

As shown in Table 1, the total score of PARBs in the CK group, the WA group, the WG group, and the WS group were 27.07, −184.64, 106.96, and 50.61, respectively. Meanwhile, the total health risk of PARBs in the CK group, WA group, WG group, and WS group were 0.067 × 10^−3^, −1.55 × 10^−3^, 87.93 × 10^−3^, and 153.53 × 10^−3^, respectively. The full information of the contribution of each factor is shown in Table S2.

**TABLE 1:** Health risk of each PARB and all PARBs in each group.

Groups	BIN ID	TFS	HR (10^−3^)	THR (10^−3^)
CK	G_bin2	6.896	0.003	0.067
	G_bin3	66.43	0.07	
	G_bin5	−4.194	0	
	G_bin8	−31.496	−0.002	
	G_bin10	−10.57	−0.004	
WA	G_bin3	−7.118	−0.068	−1.55
	G_bin4	−4.469	−0.024	
	G_bin5	−20.105	−0.324	
	G_bin6	−12.1	−0.072	
	G_bin16	−6.624	−0.091	
	G_bin21	−22.427	−0.094	
	G_bin24	−7.867	−0.056	
	G_bin30	−11.628	−0.083	
	G_bin31	−5.963	−0.018	
	G_bin33	−17.089	−0.081	
	G_bin34	−18.218	−0.13	
	G_bin38	9.455	0.068	
	G_bin39	−25.584	−0.168	
	G_bin40	−25.98	−0.356	
	G_bin46	−8.617	−0.036	
	G_bin51	−0.304	−0.015	
WG	G_bin3	7.308	17.734	87.93
	G_bin10	15.777	13.318	
	G_bin12	−11.44	−4.828	
	G_bin14	−14.394	−10.631	
	G_bin20	−6.862	−3.258	
	G_bin22	71.012	48.703	
	G_bin31	−16.805	−7.979	
	G_bin33	18.496	7.807	
	G_bin39	−21.674	−10.291	
	G_bin41	89.816	52.124	
	G_bin44	10.837	4.002	
	G_bin48	−25.407	−14.744	
	G_bin50	−7.833	−2.893	
	G_bin54	−0.317	−0.234	
	G_bin57	−1.55	−0.9	
WS	G_bin3	52.397	33.439	153.53
	G_bin4	−27.275	−5.222	
	G_bin6	−14.051	−4.185	
	G_bin7	−18.155	−4.248	
	G_bin9	−16.106	−5.139	
	G_bin12	−26.008	−3.32	
	G_bin14	5.418	1.153	
	G_bin16	−29.409	−5.631	
	G_bin20	−2.581	−0.659	
	G_bin23	−6.297	−1.875	
	G_bin24	26.49	6.762	
	G_bin25	33.181	4.235	
	G_bin27	73.005	138.22	

*Note*: “TFS” is the total factor score; “HR” is the health risk of each PARB; “THR” is the total health risks of PARBs.

The results indicated that ARGs primarily influenced the health risk of PARBs associated with factors F1, F2, F8, and F9, while VFs influenced factors F3, F4, F6, and F7. The cumulative contribution of factors F1, F2, F8, and F9 to the risk of PARBs was 39.018%, whereas factors F3, F4, F6, and F7 collectively contributed 32.720% (Table S3).

### 3.4 The effect of HD on the transfer risk of ARGs in PARBs of river sediment microbes in the reserve of wild animals

There were no ARGs carried by plasmids in the PARBs of the CK group and WS group, while there were three kinds and two kinds of PARBs containing three classes (three genes) and seven classes (eight genes) of ARGs carried by plasmids in the WA group and WG group (Table 2).

**TABLE 2:** ARGs carried by plasmids in PARBs.

Groups	BIN ID	Location	ARGs name	Antibiotic class	MGE mechanism
CK	/	/	/	/	/
WA	G_bin4	Scaffold1224	rpoB2	Multidrug	Plasmid
	G_bin16	Scaffold116	tetM	Tetracycline	Plasmid
	G_bin46	Scaffold310	floR	Phenicol	Plasmid
WG	G_bin41	Scaffold1050	dfrG	Diaminopyrimidine	Plasmid
	G_bin41	Scaffold10727	lsaE	Multidrug	Plasmid
	G_bin41	Scaffold1151	Ssui_ACT_CHL	Phenicol	Plasmid
	G_bin41	Scaffold15226	EreD	Macrolide	Plasmid
	G_bin41	Scaffold3890	aadS	Aminoglycoside	Plasmid
	G_bin41	Scaffold3890	tetX	Multidrug	Plasmid
	G_bin41	Scaffold5746	lnuG	Lincosamide	Plasmid
	G_bin57	Scaffold293	tet(C)	Tetracycline	Plasmid
WS	/	/	/	/	/

### 3.5 The effect of environmental factors on the microbial risks in the rivers

**FIGURE 5:**
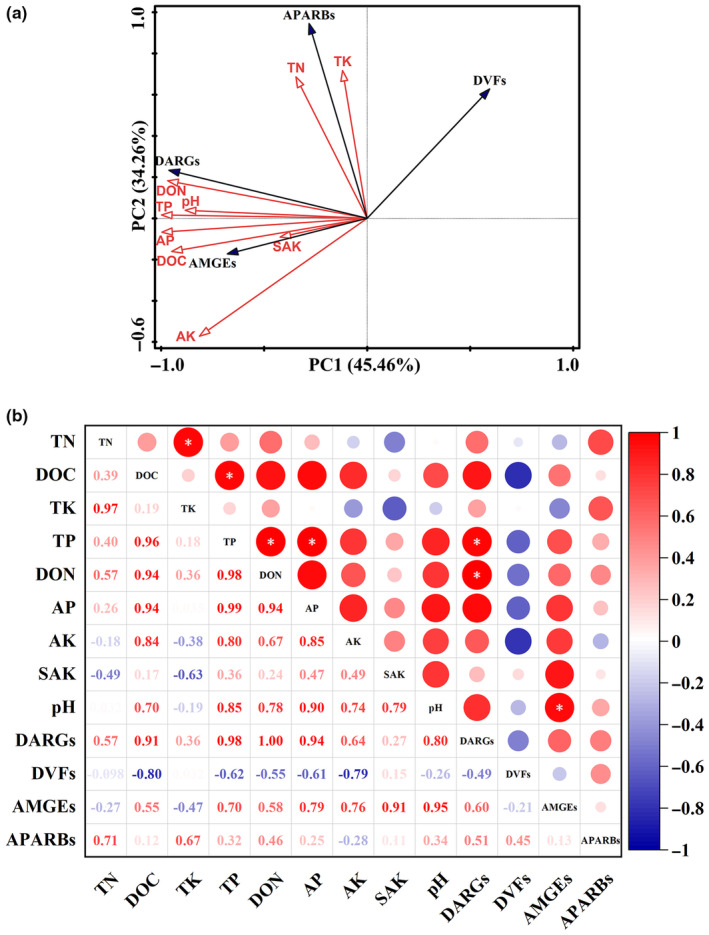
(a) The effect of various environmental factors on key microbial characteristics. The microbial characteristics included DARGs (diversity of ARGs), DVFs (diversity of VFs), AMGEs (relative abundance of MGEs), and APARBs (relative abundance of PARBs). (b) The correlation between environmental factors and microbial characteristics, where “*” indicates statistically significant correlations at *p* ≤ .05.

PCA can be used to combine the results of environmental factors and microbial risks to provide a deeper understanding of the underlying drivers of the health risks. A total of 45.46% of the variance was explained by PCA1, 34.26% by PCA2, and 79.72% by the two‐axes cumulative variance. The content of TN and TK had direct effect on PARBs, while other content of nutritious substances was mainly affected the diversity of ARGs and the relative abundance of MGEs, which exhibited positive correlations. (Figure 5a).

Further analysis using simple correlation showed that the DARGs had a significant positive correlation between the content of TP and DON (*p* < .05). pH was positively correlated with AMGEs (*p* < .05) (Figure 5b). The content of the most nutritious substances in HD groups were higher than those in the CK group (Table S4).

## 4 DISCUSSION

The higher the Simpson's index of ARGs in the environment indicates the higher the risk of ARGs to the ecological system (Yang et al., 2019). Our results showed that the Simpson's index of ARGs in rivers was increased by agriculture, grazing, and domestic sewage, which indicated that HD increased the risk of ARGs to the rivers in the habitat of wild animals. For the Simpson's index provides a comprehensive assessment by considering both the type and abundance of ARGs, and the agriculture and grazing imported new classes of ARGs to the rivers and significantly increased the total abundance of ARGs, while the domestic sewage only imported new classes of ARGs to the rivers. In other words, the significant increase in the abundance and the introduction of new types of ARGs were the reason for the increased the risk of ARGs by agriculture and grazing, while the introduction of new types of ARGs was the reason for the increased risk of ARGs by domestic sewage. Further analysis indicated that if the ARGs from the rivers under agriculture and grazing to the wild animals, then the wild animals had the potential risk of developing resistance of Sulfonamide, Beta‐lactam, Phenicol, Mupirocin, Peptide, and Nucleoside, while ARGs in the rivers under domestic sewage had the risk that made wild animals develop resistance of Sulfonamide, Beta‐lactam, Phenicol, and Mupirocin. The intrusion of HD into diverse environments contributes to heightened selective pressure on microbes, which favors the proliferation of ARGs in pathogens (Liu et al., 2021), this also indicated that HD could increase the risk of ARGs in the rivers of reserves of wild animals, and the risk of drug‐resistant pathogens to wild animals were needed further analysis.

The PARBs reflected the highest risk level of ARGs (Martínez et al., 2015). Unfortunately, PARBs were found in the rivers in the nature reserve of wild animals. The species of PARBs, as well as the total numbers of ARGs and VFs in the PARBs, were increased by HD, and some researches supported our findings (Liang et al., 2020; Zou, Xiao, et al., 2023), also indicating that HD increased the risks of rivers by increasing the kinds of PARBs, as well as their resistance and pathogenicity. Further analysis revealed that HD could increase the new types of ARGs and VFs in the PARBs. Agriculture could increase the classes of ARGs such as Fosfomycin, Fluoroquinolone antibiotic, Macrolide antibiotic, and Mupirocin, and VFs such as Biofilm, Exoenzyme, Motility, and Stress survival; grazing could increase the classes of ARGs such as Diaminopyrimidine antibiotic, Fosfomycin, Lincosamide antibiotic, Macrolide antibiotic, Phenicol antibiotic and Penam, and VFs such as Biofilm, Exoenzyme, Exotoxin, Motility and Stress survival; domestic sewage could increase the classes of VFs such as Biofilm, Exoenzyme, Exotoxin, Motility, and Stress survival.

However, the total contribution of ARGs and VFs in PARBs should be considered when the risk of PARBs in the ecological environment is evaluated. Several studies using factor analysis had shown that the higher contribution of ARGs and VFs in a PARB lead to the higher the health risk of the PARB (Bai et al., 2020; Zou, Yuan, et al., 2023). Our results indicated that grazing and domestic sewage could enhance the contribution of ARGs and VFs to the risk of PARBs in rivers, and grazing had the greatest contribution to the risk of PARBs. Moreover, the risk of a PARB to the environment was also affected by its abundance (Liu et al., 2021). It was found that the total relative abundance of PARBs in the river sediments under grazing and domestic sewage was higher than that without HD, resulting in the higher total health risks of PARBs under grazing and domestic sewage. The results also showed that the contribution of the factors belonged to ARGs was higher than that belonged to VFs, indicating that ARGs contributed more to the health risk of PARBs than VFs.

Overall, grazing and domestic sewage could increase the risk of ARGs in PARBs to the rivers, which was mainly reflected in two aspects. On the one hand, the pathogenic mechanisms and the drug resistance scope of the PARBs were increased by grazing and domestic sewage. On the other hand, the risks of multidrug PARBs were increased by grazing and domestic sewage. Multidrug PARBs carrying multidrug resistance genes pose a severe threat to public health risks and increase the risk of superbugs (Zhao et al., 2020), and the kinds of multidrug PARBs in the WG group and WS group were more than that in the CK group. These two aspects indicated that the risk of pathogenic infections in wild animals increased, and the scope of antibiotic resistance will be greatly expanded if wild animals are treated with antibiotics.

In summary, grazing and domestic sewage increased the serious potential risks of PARBs to rivers in the reserve of wild animals. If these PARBs enter wild animal populations through rivers, they may cause extreme health hazards to wild animals and may not be treated well with antibiotics. But, we surprisingly found that although agriculture could increase the total abundance of ARGs, it was determined to reduce the risk of PARBs. The risk of PARBs in the WA group could be diluted by other bacteria, the low contribution of ARGs and VFs to the risk of PARBs seems to confirm that. However, it also indicated a potential problem that non‐pathogenic and opportunistic bacteria might carry a large number of ARGs. Although these bacteria did not significantly increase the risk of PARBs, most studies have shown that the microbial risks posed by these bacteria cannot be ignored (Alexander et al., 2015; Bouki et al., 2013; Serwecińska, 2020).

MGEs play a pivotal role in the horizontal transfer of genes (Kumkar et al., 2022). Plasmids are particularly important MGEs, serve as crucial carriers for the transmission of ARGs (Zhu et al., 2023). The horizontal transfer of ARGs through plasmids increases the risk of superbug in the environment (Pan et al., 2020). Our study indicated that the PARBs under all kinds of HD had the risk from the rivers to wild animals in the nature reserve of wild animals, and agriculture and grazing increased the transfer risks of ARGs in PARBs to wild animals because agriculture and grazing increased the total numbers of ARGs carried by plasmids in PARBs. Our results indicated that the transfer risks of ARGs in PARBs under grazing was the highest, for the gene numbers of ARGs were the highest, as well as the resistance scopes were the widest. In addition, it is noteworthy that most of the transferable ARGs in the WG group came from G_bin41, so we believe that the monitoring of this PARB should be strengthened. Fortunately, our results also indicated an optimistic result that not all classes of ARGs had the risk of horizontal transfer to wild animals. Because we found that among the 16 classes of ARGs detected in the habitats of wild animals, only seven classes of ARGs are at the risk of horizontal transfer to wild animals, and the ARGs classes of Aminocoumarin, Fosfomycin, Glycopeptide, Rifamycin, Fosfomycin, Fluoroquinolone, Mupirocin, Nitroimidazole, and Penam had no transfer risks from the rivers to wild animals. Overall, HD enhanced the transfer risk of ARGs through plasmids. Therefore, it was urgent to study what were the driving factors that enhanced the transfer risk of ARGs from human‐disturbed habitats to wild animals.

Environmental factors are key drivers of microbial risks (Chen et al., 2021). Based on PCA analysis, we found that the environmental factors also had impacts on the risk of PARBs. Specifically, the content of TN and TK would mainly affect the risks of PARBs by affecting the relative abundance of PARBs, while the content of DON, TP, AP, DOC, SAK, and AK, and pH affecting the diversity and transfer ability of ARGs, our results were supported by some previous researches (Liu et al., 2021; Zhang et al., 2018). Unfortunately, we found that the agriculture, grazing and domestic sewage could increase the content of nutritious substances. Once the contaminants from HD enter the water, they probably have various effects on the risk of ARGs (Boto et al., 2019), which supported our results that HD could increase the risk of ARGs by increasing the content of nutritious substances. Further analysis found that pH was positively correlated with the abundance of MGEs, and Xu et al. (Xu et al., 2022) showed that the high pH might promote the spread of ARGs in the environment, which indicated that the higher the pH, the higher the possibility of MGEs to transfer ARGs. Both of the pH under agriculture and grazing was higher than wild habitat, while the pH under domestic sewage was lower than wild habitat, for domestic sewage may contain some substances such as cellulose, starch, sugars, fatty, and proteins, which could produce acidic substances such as hydrogen sulfide and volatile fatty acids, phosphoric acid, and carbonic acid in the anaerobic environment of river sediments (Capone & Kiene, 1988; Huang et al., 2010). The lower pH could also be used to explain why MGEs were not checked under domestic sewage.

In addition, the content of TP and DON were positively correlated with the diversity of ARGs, which indicated that the higher content of TP or DON could promote an increase in the higher risk of ARGs. However, VFs did not significantly correlate with other environmental factors, which indicated that the contamination from HD increased health risk of rivers by increasing the risk of ARGs rather than the risk of VFs. There was also a significant correlation between the content of TP and DON, which indicated that these two nutrients would have a synergistic effect to enhance the risk of ARGs. These results could be explained in water environments, and it is noteworthy that water environments are forced to receive amounts of nutritious substances from HD (Boxall et al., 2012). Our study released a significant health concern to protect wild animals. Nitrogen and phosphorus are the main indicators of water eutrophication (Conley et al., 2009). Agriculture, grazing, and domestic sewage are the main sources of nitrogen and phosphorus to rivers (Eliassen & Tchobanoglous, 1969; Nelson et al., 1996; Sharpley et al., 1987), and our result showed that HD increased the content of nitrogen and phosphorus. Thus, we suggest that grazing manure could be collected or buried with soil. Composting has been shown by most studies to reduce the microbial risks (Gou et al., 2018; Singh & Nain, 2014), the domestic waste and feces could be composted for agriculture, which would reduce the need for chemical fertilizers and reduce the input of nitrogen and phosphorus from agriculture and domestic sewage into rivers. To achieve these practices, it is necessary to focus on the planning of production activities, rigorously managing wastewater discharge methods, and promptly implementing measures to improve wastewater treatment protocols. Most importantly, we emphasized while protecting wild animals, we must strengthen to monitor the health risk of rivers in nature reserves of wild animals, especially the risk of ARGs.

## 5 CONCLUSION

It was determined that HD increased the diversity of ARGs in river sediments in the nature reserve of wild animals. We found that grazing and domestic sewage increased the health risk of rivers, and the risk of ARGs in PARBs being transferred to wild animals under grazing was higher than that of wild habitat. The total health risks of agriculture were lower than that of wild habitat, but agriculture introduced new pathogenic mechanisms and increased the range of resistance to PARBs, and enhanced the potential risk of transfer of ARGs in PARBs. Therefore, agriculture, grazing, and domestic sewage increased the health risk of PARBs in rivers of the nature reserve of wild animals, and agriculture and grazing increased the risk of ARGs in PARBs being transferred to wild animals. Among them, domestic sewage posed the greatest health risk to rivers, but grazing posed the greatest risk of ARGs being transferred from PARBs in the rivers to wild animals. The contribution of ARGs to river health was greater than that of VFs. HD mainly increased the risk of ARGs to increase the health risk of rivers by discharging nitrogen and phosphorus into rivers. Therefore, we suggest that under the premise that HD for survival cannot be avoided, we should control the anthropogenic discharge of nitrogen and phosphorus into rivers, and detect the content of nitrogen and phosphorus, so as to control the risk of ARGs from HD to river health.

## AUTHOR CONTRIBUTIONS

**Rongpan Hu:** Data curation (equal); investigation (equal); methodology (equal); software (equal); validation (equal); writing – original draft (equal). **Minxing Ren:** Formal analysis (equal); investigation (equal); validation (equal); visualization (equal). **Sumei Liang:** Investigation (equal); methodology (equal); validation (equal). **Shuzhen Zou:** Funding acquisition (equal); methodology (equal); supervision (equal); writing – review and editing (equal). **Dayong Li:** Data curation (equal); funding acquisition (equal); supervision (equal); validation (equal); writing – review and editing (equal).

## CONFLICT OF INTEREST STATEMENT

None declared.

## Supporting information


Appendix S1

## Data Availability

The datasets presented in this study can be found in online repositories. The names of the repository/repositories and accession number(s) can be found at: https://submit.ncbi.nlm.nih.gov/subs/sra/SUB13994222/overview, PRJNA1044275.

## References

[ece311435-bib-0001] Abdugheni, R. , Li, L. , Yang, Z.‐N. , Huang, Y. , Fang, B.‐Z. , Shurigin, V. , Mohamad, O. A. , Liu, Y.‐H. , & Li, W.‐J. (2023). Microbial risks caused by livestock excrement: Current research status and prospects. Microorganisms, 11, 1897. 10.3390/microorganisms11081897 37630456 PMC10456746

[ece311435-bib-0002] Acheampong, A. O. , & Opoku, E. E. O. (2023). Environmental degradation and economic growth: Investigating linkages and potential pathways. Energy Economics, 123, 106734.

[ece311435-bib-0003] Alexander, J. , Bollmann, A. , Seitz, W. , & Schwartz, T. (2015). Microbiological characterization of aquatic microbiomes targeting taxonomical marker genes and antibiotic resistance genes of opportunistic bacteria. The Science of the Total Environment, 512, 316–325. 25634736 10.1016/j.scitotenv.2015.01.046

[ece311435-bib-0004] Bai, Y. , Wang, Q. , Liang, J. , Liao, K. , & Qu, J. (2020). A method for evaluating water health risks based on resistance genes and virulence factor genes. National Invention Patent.

[ece311435-bib-0005] Bankevich, A. , Nurk, S. , Antipov, D. , Gurevich, A. A. , Dvorkin, M. , Kulikov, A. S. , Lesin, V. M. , Nikolenko, S. I. , Pham, S. , Prjibelski, A. D. , Pyshkin, A. V. , Sirotkin, A. V. , Vyahhi, N. , Tesler, G. , Alekseyev, M. A. , & Pevzner, P. A. (2012). SPAdes: A new genome assembly algorithm and its applications to single‐cell sequencing. Journal of Computational Biology, 19(5), 455–477. 22506599 10.1089/cmb.2012.0021 PMC3342519

[ece311435-bib-0006] Boto, L. , Pineda, M. , & Pineda, R. (2019). Potential impacts of horizontal gene transfer on human health and physiology and how anthropogenic activity can affect it. The FEBS Journal, 286(20), 3959–3967. 31495055 10.1111/febs.15054

[ece311435-bib-0007] Bouki, C. , Venieri, D. , & Diamadopoulos, E. (2013). Detection and fate of antibiotic resistant bacteria in wastewater treatment plants: A review. Ecotoxicology and Environmental Safety, 91, 1–9. 23414720 10.1016/j.ecoenv.2013.01.016

[ece311435-bib-0008] Boxall, A. B. , Rudd, M. A. , Brooks, B. W. , Caldwell, D. J. , Choi, K. , Hickmann, S. , Innes, E. , Ostapyk, K. , Staveley, J. P. , Verslycke, T. , Ankley, G. T. , Beazley, K. F. , Belanger, S. E. , Berninger, J. P. , Carriquiriborde, P. , Coors, A. , Deleo, P. C. , Dyer, S. D. , Ericson, J. F. , … Van Der Kraak, G. (2012). Pharmaceuticals and personal care products in the environment: What are the big questions? Environmental Health Perspectives, 120(9), 1221–1229. 22647657 10.1289/ehp.1104477 PMC3440110

[ece311435-bib-0009] Capone, D. G. , & Kiene, R. P. (1988). Comparison of microbial dynamics in marine and freshwater sediments: Contrasts in anaerobic carbon catabolism 1. Limnology and Oceanography, 33(4part2), 725–749.

[ece311435-bib-0010] Chaumeil, P.‐A. , Mussig, A. J. , Hugenholtz, P. , & Parks, D. H. (2019). GTDB‐Tk: A toolkit to classify genomes with the genome taxonomy database. Bioinformatics, 36(6), 1925–1927. 31730192 10.1093/bioinformatics/btz848 PMC7703759

[ece311435-bib-0011] Chen, B. , Hao, L. , Guo, X. , Wang, N. , & Ye, B. (2015). Prevalence of antibiotic resistance genes of wastewater and surface water in livestock farms of Jiangsu Province, China. Environmental Science and Pollution Research, 22(18), 13950–13959. 25948386 10.1007/s11356-015-4636-y

[ece311435-bib-0012] Chen, H. , Liu, C. , Teng, Y. , Zhang, Z. , Chen, Y. , & Yang, Y. (2021). Environmental risk characterization and ecological process determination of bacterial antibiotic resistome in lake sediments. Environment International, 147, 106345. 33385921 10.1016/j.envint.2020.106345

[ece311435-bib-0013] Cheng, W. , Chen, H. , Su, C. , & Yan, S. (2013). Abundance and persistence of antibiotic resistance genes in livestock farms: A comprehensive investigation in eastern China. Environment International, 61, 1–7. 24091253 10.1016/j.envint.2013.08.023

[ece311435-bib-0014] Conley, D. J. , Paerl, H. W. , Howarth, R. W. , Boesch, D. F. , Seitzinger, S. P. , Havens, K. E. , Lancelot, C. , & Likens, G. E. J. S. (2009). Controlling eutrophication: Nitrogen and phosphorus (pp. 1014–1015). American Association for the Advancement of Science. 10.1126/science.1167755 19229022

[ece311435-bib-0015] Czekalski, N. , Sigdel, R. , Birtel, J. , Matthews, B. , & Bürgmann, H. (2015). Does human activity impact the natural antibiotic resistance background? Abundance of antibiotic resistance genes in 21 Swiss lakes. Environment International, 81, 45–55. 25913323 10.1016/j.envint.2015.04.005

[ece311435-bib-0016] Doherty, T. S. , Hays, G. C. , & Driscoll, D. A. (2021). Human disturbance causes widespread disruption of animal movement. Nature Ecology & Evolution, 5(4), 513–519. 33526889 10.1038/s41559-020-01380-1

[ece311435-bib-0017] Eliassen, R. , & Tchobanoglous, G. (1969). Removal of nitrogen and phosphorus from waste water. Environmental Science & Technology, 3(6), 536–541.

[ece311435-bib-0018] Fackelmann, G. , Gillingham, M. A. F. , Schmid, J. , Heni, A. C. , Wilhelm, K. , Schwensow, N. , & Sommer, S. (2021). Human encroachment into wildlife gut microbiomes. Communications Biology, 4(1), 800. 34172822 10.1038/s42003-021-02315-7 PMC8233340

[ece311435-bib-0019] Gou, M. , Hu, H.‐W. , Zhang, Y.‐J. , Wang, J.‐T. , Hayden, H. , Tang, Y.‐Q. , & He, J.‐Z. (2018). Aerobic composting reduces antibiotic resistance genes in cattle manure and the resistome dissemination in agricultural soils. The Science of the Total Environment, 612, 1300–1310. 28898936 10.1016/j.scitotenv.2017.09.028

[ece311435-bib-0020] Gu, Z. , Gong, J. , & Wang, Y. (2023). Construction and evaluation of ecological networks among natural protected areas based on “quality‐structure–function”: A case study of the Qinghai‐Tibet area. Ecological Indicators, 151, 110228.

[ece311435-bib-0021] Guo, Q. , & Wu, K. (2023). Study on the mechanism of benefit distribution among government, enterprises and indigenous people in natural heritage reserves. Journal of Cleaner Production, 415, 137828.

[ece311435-bib-0022] Huang, M.‐H. , Li, Y.‐M. , & Gu, G.‐W. (2010). Chemical composition of organic matters in domestic wastewater. Desalination, 262(1–3), 36–42.

[ece311435-bib-0023] Jeon, J. H. , Jang, K.‐M. , Lee, J. H. , Kang, L.‐W. , & Lee, S. H. (2023). Transmission of antibiotic resistance genes through mobile genetic elements in *Acinetobacter baumannii* and gene‐transfer prevention. Science of the Total Environment, 857, 159497. 36257427 10.1016/j.scitotenv.2022.159497

[ece311435-bib-0024] Jokanović, S. , Kajan, K. , Perović, S. , Ivanić, M. , Mačić, V. , & Orlić, S. (2021). Anthropogenic influence on the environmental health along Montenegro coast based on the bacterial and chemical characterization. Environmental Pollution, 271, 116383. 33387780 10.1016/j.envpol.2020.116383

[ece311435-bib-0025] Kang, D. D. , Li, F. , Kirton, E. , Thomas, A. , Egan, R. , An, H. , & Wang, Z. (2019). MetaBAT 2: An adaptive binning algorithm for robust and efficient genome reconstruction from metagenome assemblies. PeerJ, 7, e7359. 31388474 10.7717/peerj.7359 PMC6662567

[ece311435-bib-0026] Khan, H. , Miao, X. , Liu, M. , Ahmad, S. , & Bai, X. (2020). Behavior of last resort antibiotic resistance genes (Mcr‐1 and blaNDM‐1) in a drinking water supply system and their possible acquisition by the mouse gut flora. Environmental pollution (Barking, Essex: 1987), 259, 113818. 31896482 10.1016/j.envpol.2019.113818

[ece311435-bib-0027] Kim, H. , Kim, M. , Kim, S. , Lee, Y. M. , & Shin, S. C. (2022). Characterization of antimicrobial resistance genes and virulence factor genes in an Arctic permafrost region revealed by metagenomics. Environmental Pollution, 294, 118634. 34875269 10.1016/j.envpol.2021.118634

[ece311435-bib-0028] Kumkar, S. N. , Kamble, E. E. , Chavan, N. S. , Dhotre, D. P. , & Pardesi, K. R. (2022). Diversity of resistant determinants, virulence factors, and mobile genetic elements in *Acinetobacter baumannii* from India: A comprehensive in silico genome analysis. Frontiers in Cellular and Infection Microbiology, 12, 997897. 36519127 10.3389/fcimb.2022.997897 PMC9742364

[ece311435-bib-0029] Lamberte, L. E. , & van Schaik, W. (2022). Antibiotic resistance in the commensal human gut microbiota. Current Opinion in Microbiology, 68, 102150. 35490629 10.1016/j.mib.2022.102150

[ece311435-bib-0030] Li, D. , Liu, C.‐M. , Luo, R. , Sadakane, K. , & Lam, T.‐W. (2015). MEGAHIT: An ultra‐fast single‐node solution for large and complex metagenomics assembly via succinct de Bruijn graph. Bioinformatics, 31(10), 1674–1676. 25609793 10.1093/bioinformatics/btv033

[ece311435-bib-0031] Li, Z. , Li, T. , Xing, X. , Bi, Z. , Qi, P. , Hu, C. , Xu, G. , Chen, C. , Ma, K. , & Chen, J. (2022). Inhibiting the increase of antibiotic resistance genes during drinking water distribution by superior microbial interface using Fe modified granular activated carbon. Journal of Cleaner Production, 335, 130225.

[ece311435-bib-0032] Liang, J. , Mao, G. , Yin, X. , Ma, L. , Liu, L. , Bai, Y. , Zhang, T. , & Qu, J. (2020). Identification and quantification of bacterial genomes carrying antibiotic resistance genes and virulence factor genes for aquatic microbiological risk assessment. Water Research, 168, 115160. 31614233 10.1016/j.watres.2019.115160

[ece311435-bib-0033] Liu, S. , Wang, P. , Wang, C. , Wang, X. , & Chen, J. (2021). Anthropogenic disturbances on antibiotic resistome along the Yarlung Tsangpo River on the Tibetan Plateau: Ecological dissemination mechanisms of antibiotic resistance genes to bacterial pathogens. Water Research, 202, 117447. 34325101 10.1016/j.watres.2021.117447

[ece311435-bib-0034] Lu, L. , He, Y. , Peng, C. , Wen, X. , Ye, Y. , Ren, D. , Tang, Y. , & Zhu, D. (2022). Dispersal of antibiotic resistance genes in an agricultural influenced multi‐branch river network. Science of the Total Environment, 830, 154739. 35331763 10.1016/j.scitotenv.2022.154739

[ece311435-bib-0035] Luo, J.‐C. , Long, H. , Zhang, J. , Zhao, Y. , & Sun, L. (2021). Characterization of a deep sea *Bacillus toyonensis* isolate: Genomic and pathogenic features. Frontiers in Cellular and Infection Microbiology, 11, 629116. 33777842 10.3389/fcimb.2021.629116 PMC7988205

[ece311435-bib-0036] Ma, L. , Xia, Y. , Li, B. , Yang, Y. , Li, L.‐G. , Tiedje, J. M. , & Zhang, T. (2016). Metagenomic assembly reveals hosts of antibiotic resistance genes and the shared resistome in pig, chicken, and human feces. Environmental Science & Technology, 50(1), 420–427. 26650334 10.1021/acs.est.5b03522

[ece311435-bib-0037] Marti, E. , Variatza, E. , & Balcazar, J. L. (2014). The role of aquatic ecosystems as reservoirs of antibiotic resistance. Trends in Microbiology, 22(1), 36–41. 24289955 10.1016/j.tim.2013.11.001

[ece311435-bib-0038] Martínez, J. L. , Coque, T. M. , & Baquero, F. (2015). What is a resistance gene? Ranking risk in resistomes. Nature Reviews. Microbiology, 13(2), 116–123. 25534811 10.1038/nrmicro3399

[ece311435-bib-0039] Miller, T. K. , Pierce, K. , Clark, E. E. , & Primack, R. B. (2023). Wildlife rehabilitation records reveal impacts of anthropogenic activities on wildlife health. Biological Conservation, 286, 110295.

[ece311435-bib-0040] Mware, N. A. , Hall, M. C. , Rajendran, S. , Gilley, J. E. , Schmidt, A. M. , Bartelt‐Hunt, S. L. , Zhang, Y. , & Li, X. (2022). Resistome and mobilome in surface runoff from manured soil as affected by setback distance. Journal of Hazardous Materials, 429, 128278. 35065306 10.1016/j.jhazmat.2022.128278

[ece311435-bib-0041] Na, G. , Lu, Z. , Gao, H. , Zhang, L. , Li, Q. , Li, R. , Yang, F. , Huo, C. , & Yao, Z. (2018). The effect of environmental factors and migration dynamics on the prevalence of antibiotic‐resistant *Escherichia coli* in estuary environments. Scientific Reports, 8(1), 1663. 29374235 10.1038/s41598-018-20077-x PMC5786026

[ece311435-bib-0042] Nelson, P. N. , Cotsaris, E. , & Oades, J. M. (1996). Nitrogen, phosphorus, and organic carbon in streams draining two grazed catchments. Wiley Online Library.

[ece311435-bib-0043] Oksanen, J. , Blanchet, F. G. , Kindt, R. , Legendre, P. , Minchin, P. , O'hara, R. , Simpson, G. , Solymos, P. , Stevens, M. , & Wagner, H. (2017). Vegan: Community ecology package. R package (pp. 25–27). University of Helsinki.

[ece311435-bib-0044] Olsen, S. R. (1954). Estimation of available phosphorus in soils by extraction with sodium bicarbonate. US Department of Agriculture.

[ece311435-bib-0045] Pan, Y. , Zeng, J. , Li, L. , Yang, J. , Tang, Z. , Xiong, W. , Li, Y. , Chen, S. , & Zeng, Z. (2020). Coexistence of antibiotic resistance genes and virulence factors deciphered by large‐scale complete genome analysis. mSystems, 5(3), e00821‐19. 10.1128/msystems.00821-19 PMC8534731 32487745

[ece311435-bib-0046] Pruden, A. , Arabi, M. , & Storteboom, H. N. (2012). Correlation between upstream human activities and riverine antibiotic resistance genes. Environmental Science & Technology, 46(21), 11541–11549. 23035771 10.1021/es302657r

[ece311435-bib-0047] Qiu, J. (2015). Experts question China's panda survey. Nature News, 10720. 10.1038/nature.2015.17020

[ece311435-bib-0048] Rolain, J.‐M. (2013). Food and human gut as reservoirs of transferable antibiotic resistance encoding genes. Frontiers in Microbiology, 4, 173. 23805136 10.3389/fmicb.2013.00173 PMC3690338

[ece311435-bib-0049] Rout, A. K. , Das, R. , Mahanandia, N. C. , Dey, S. , Parida, S. N. , Mondal, M. , Panda, S. P. , Jena, R. , Behera, B. , & Behera, B. K. (2023). Identifying novel antibiotic resistance genes (ARGs): Important aspect of metagenomic research. In B. K. Behera (Ed.), Biotechnological tools in fisheries and aquatic health management (pp. 231–246). Springer. 10.1007/978-981-99-2981-8_12

[ece311435-bib-0050] Rout, A. K. , Tripathy, P. S. , Dixit, S. , Behera, D. U. , Behera, B. , Das, B. K. , & Behera, B. K. (2023). Unveiling the microbiome landscape: A metagenomic study of bacterial diversity, antibiotic resistance, and virulence factors in the sediments of the river ganga, India. Antibiotics (Basel, Switzerland), 12(12), 1735. 38136769 10.3390/antibiotics12121735 PMC10740832

[ece311435-bib-0051] Rout, A. K. , Tripathy, P. S. , Dixit, S. , Behera, D. U. , Behera, B. , Das, B. K. , & Behera, B. K. (2024). Metagenomics analysis of sediments of river Ganga, India for bacterial diversity, functional genomics, antibiotic resistant genes and virulence factors. Current Research in Biotechnology, 7, 100187.

[ece311435-bib-0052] Serwecińska, L. (2020). Antimicrobials and antibiotic‐resistant bacteria: A risk to the environment and to public health. Water, 12(12), 3313.

[ece311435-bib-0053] Shao, S. , Hu, Y. , Cheng, J. , & Chen, Y. (2018). Research progress on distribution, migration, transformation of antibiotics and antibiotic resistance genes (ARGs) in aquatic environment. Critical Reviews in Biotechnology, 38(8), 1195–1208. 29807455 10.1080/07388551.2018.1471038

[ece311435-bib-0054] Sharpley, A. N. , Smith, S. , & Naney, J. (1987). Environmental impact of agricultural nitrogen and phosphorus use. Journal of Agricultural and Food Chemistry, 35(5), 812–817.

[ece311435-bib-0055] Shi, Y. , Li, Y. , Xiang, X. , Sun, R. , Yang, T. , He, D. , Zhang, K. , Ni, Y. , Zhu, Y.‐G. , Adams, J. M. , & Chu, H. (2018). Spatial scale affects the relative role of stochasticity versus determinism in soil bacterial communities in wheat fields across the North China plain. Microbiome, 6(1), 27. 29402331 10.1186/s40168-018-0409-4 PMC5799910

[ece311435-bib-0056] Singh, S. , & Nain, L. (2014). Microorganisms in the conversion of agricultural wastes to compost. Proceedings of the Indian National Science Academy, 80, 473–481.

[ece311435-bib-0057] Tan, R. , Jin, M. , Chen, Z. , Shao, Y. , Song, Y. , Yin, J. , Wang, L. , Chen, T. , Li, J. , & Yang, D. (2023). Exogenous antibiotic resistance gene contributes to intestinal inflammation by modulating the gut microbiome and inflammatory cytokine responses in mouse. Gut Microbes, 15(1), 2156764. 36573825 10.1080/19490976.2022.2156764 PMC9809935

[ece311435-bib-0058] Uritskiy, G. V. , DiRuggiero, J. , & Taylor, J. (2018). MetaWRAP—A flexible pipeline for genome‐resolved metagenomic data analysis. Microbiome, 6(1), 158. 30219103 10.1186/s40168-018-0541-1 PMC6138922

[ece311435-bib-0059] van der Graaf‐van Bloois, L. , Wagenaar, J. A. , & Zomer, A. L. (2021). RFPlasmid: Predicting plasmid sequences from short‐read assembly data using machine learning. Microbial Genomics, 7(11), 683. 10.1099/mgen.0.000683 PMC8743549 34846288

[ece311435-bib-0060] Wang, H. , Liu, X. , Zhao, C. , Chang, Y. , Liu, Y. , & Zang, F. (2021). Spatial‐temporal pattern analysis of landscape ecological risk assessment based on land use/land cover change in Baishuijiang national nature reserve in Gansu Province, China. Ecological Indicators, 124, 107454.

[ece311435-bib-0061] Wang, J. , Fan, H. , He, X. , Zhang, F. , Xiao, J. , Yan, Z. , Feng, J. , & Li, R. (2021). Response of bacterial communities to variation in water quality and physicochemical conditions in a river‐reservoir system. Global Ecology and Conservation, 27, e01541.

[ece311435-bib-0062] Wu, C. , Zhu, R. , Lu, Y. , Li, D. , Xiao, Y. , Cui, W. , & Li, N. (2023). Editorial: Three‐way interactions between host, environment, and microbiome: Importance of microbiology in the one health. Frontiers in Microbiology, 14, 1177119. 37025636 10.3389/fmicb.2023.1177119 PMC10071026

[ece311435-bib-0063] Wu, D. , Jin, L. , Xie, J. , Liu, H. , Zhao, J. , Ye, D. , & Li, X.‐D. (2022). Inhalable antibiotic resistomes emitted from hospitals: Metagenomic insights into bacterial hosts, clinical relevance, and environmental risks. Microbiome, 10(1), 19. 35086564 10.1186/s40168-021-01197-5 PMC8796446

[ece311435-bib-0064] Xu, Y. , You, G. , Zhang, M. , Peng, D. , Jiang, Z. , Qi, S. , Yang, S. , & Hou, J. (2022). Antibiotic resistance genes alternation in soils modified with neutral and alkaline salts: Interplay of salinity stress and response strategies of microbes. The Science of the Total Environment, 809, 152246. 34896144 10.1016/j.scitotenv.2021.152246

[ece311435-bib-0065] Yang, Y. , Liu, G. , Ye, C. , & Liu, W. (2019). Bacterial community and climate change implication affected the diversity and abundance of antibiotic resistance genes in wetlands on the Qinghai‐Tibetan plateau. Journal of Hazardous Materials, 361, 283–293. 30212791 10.1016/j.jhazmat.2018.09.002

[ece311435-bib-0066] Zeng, J. , Pan, Y. , Yang, J. , Hou, M. , Zeng, Z. , & Xiong, W. (2019). Metagenomic insights into the distribution of antibiotic resistome between the gut‐associated environments and the pristine environments. Environment International, 126, 346–354. 30826613 10.1016/j.envint.2019.02.052

[ece311435-bib-0067] Zhang, A.‐N. , Gaston, J. M. , Dai, C. L. , Zhao, S. , Poyet, M. , Groussin, M. , Yin, X. , Li, L.‐G. , van Loosdrecht, M. C. , & Topp, E. (2021). An omics‐based framework for assessing the health risk of antimicrobial resistance genes. Nature Communications, 12(1), 4765. 10.1038/s41467-021-25096-3 PMC8346589 34362925

[ece311435-bib-0068] Zhang, B. , Qin, S. , Guan, X. , Jiang, K. , Jiang, M. , & Liu, F. (2021). Distribution of antibiotic resistance genes in Karst River and its ecological risk. Water Research, 203, 117507. 34392041 10.1016/j.watres.2021.117507

[ece311435-bib-0069] Zhang, L. , Pan, B. , Jiang, X. , Wang, H. , Lu, Y. , Lu, Y. , & Li, R. (2020). Responses of the macroinvertebrate taxonomic distinctness indices of lake fauna to human disturbances in the middle and lower reaches of the Yangtze river. Ecological Indicators, 110, 105952.

[ece311435-bib-0070] Zhang, L. , Xu, W.‐H. , Ouyang, Z.‐Y. , & Zhu, C.‐Q. (2014). Determination of priority nature conservation areas and human disturbances in the Yangtze River basin, China. Journal for Nature Conservation, 22(4), 326–336.

[ece311435-bib-0071] Zhang, Q.‐Q. , Tian, G.‐M. , & Jin, R.‐C. (2018). The occurrence, maintenance, and proliferation of antibiotic resistance genes (ARGs) in the environment: Influencing factors, mechanisms, and elimination strategies. Applied Microbiology and Biotechnology, 102, 8261–8274. 30056512 10.1007/s00253-018-9235-7

[ece311435-bib-0072] Zhang, X. , Johnston, E. R. , Barberán, A. , Ren, Y. , Lü, X. , & Han, X. (2017). Decreased plant productivity resulting from plant group removal experiment constrains soil microbial functional diversity. Global Change Biology, 23(10), 4318–4332. 28585356 10.1111/gcb.13783

[ece311435-bib-0073] Zhang, Z. , Zhang, Q. , Wang, T. , Xu, N. , Lu, T. , Hong, W. , Penuelas, J. , Gillings, M. , Wang, M. , & Gao, W. (2022). Assessment of global health risk of antibiotic resistance genes. Nature Communications, 13(1), 1553. 10.1038/s41467-022-29283-8 PMC8943045 35322038

[ece311435-bib-0074] Zhao, H. , Zhang, J. , Chen, X. , Yang, S. , Huang, H. , Pan, L. , Huang, L. , Jiang, G. , Tang, J. , & Xu, Q. (2023). Climate and nutrients regulate biographical patterns and health risks of antibiotic resistance genes in mangrove environment. The Science of the Total Environment, 854, 158811. 36115398 10.1016/j.scitotenv.2022.158811

[ece311435-bib-0075] Zhao, Z. , Zhang, K. , Wu, N. , Li, W. , Xu, W. , Zhang, Y. , & Niu, Z. (2020). Estuarine sediments are key hotspots of intracellular and extracellular antibiotic resistance genes: A high‐throughput analysis in Haihe Estuary in China. Environment International, 135, 105385. 31855802 10.1016/j.envint.2019.105385

[ece311435-bib-0076] Zhou, Z.‐C. , Zheng, J. , Wei, Y.‐Y. , Chen, T. , Dahlgren, R. A. , Shang, X. , & Chen, H. (2017). Antibiotic resistance genes in an urban river as impacted by bacterial community and physicochemical parameters. Environmental Science and Pollution Research International, 24, 23753–23762. 28864929 10.1007/s11356-017-0032-0

[ece311435-bib-0077] Zhu, D. , Lu, L. , Zhang, Z. , Qi, D. , Zhang, M. , O'Connor, P. , Wei, F. , & Zhu, Y.‐G. (2021). Insights into the roles of fungi and protist in the giant panda gut microbiome and antibiotic resistome. Environment International, 155, 106703. 34139588 10.1016/j.envint.2021.106703

[ece311435-bib-0078] Zhu, W. , Lomsadze, A. , & Borodovsky, M. (2010). Ab initio gene identification in metagenomic sequences. Nucleic Acids Research, 38(12), e132. 20403810 10.1093/nar/gkq275 PMC2896542

[ece311435-bib-0079] Zhu, Y. , Liu, Z. , Hu, B. , & Zhu, L. (2023). Partitioning and migration of antibiotic resistance genes at soil‐water‐air interface mediated by plasmids. Environmental Pollution, 327, 121557. 37019265 10.1016/j.envpol.2023.121557

[ece311435-bib-0080] Zinsstag, J. , Kaiser‐Grolimund, A. , Heitz‐Tokpa, K. , Sreedharan, R. , Lubroth, J. , Caya, F. , Stone, M. , Brown, H. , Bonfoh, B. , Dobell, E. , Morgan, D. , Homaira, N. , Kock, R. , Hattendorf, J. , Crump, L. , Mauti, S. , del Rio Vilas, V. , Saikat, S. , Zumla, A. , … de la Rocque, S. (2023). Advancing one human–animal–environment health for global health security: What does the evidence say? The Lancet, 401(10376), 591–604. 10.1016/S0140-6736(22)01595-1 36682371

[ece311435-bib-0081] Zou, S. , Yuan, T. , Lu, T. , Yan, J. , Kang, D. , & Li, D. (2023). Human disturbance increases health risks to golden snub‐nosed monkeys and the transfer risk of pathogenic antibiotic‐resistant bacteria from golden snub‐nosed monkeys to humans. Animals (Basel), 13(19), 3083. 37835689 10.3390/ani13193083 PMC10572025

[ece311435-bib-0082] Zou, Y. , Xiao, Z. , Wang, L. , Wang, Y. , Yin, H. , & Li, Y. (2023). Prevalence of antibiotic resistance genes and virulence factors in the sediment of WWTP effluent‐dominated rivers. Science of the Total Environment, 897, 165441. 37437635 10.1016/j.scitotenv.2023.165441

