# Supplementary material for: Effects of antibiotic resistance genes on health risks of rivers in habitat of wild animals under human disturbance – based on analysis of antibiotic resistance genes and virulence factors in microbes of river sediments
Source: Ecol Evol. 2024 May 24;14(5):e11435. doi: 10.1002/ece3.11435 (PMC11126646; doi:10.1002/ece3.11435)
Supplement: Supplementary file 1 — Appendix S1 [file ECE3-14-e11435-s001.docx]

Table S1 Characteristics of high-quality metagenomic assembly genomes (MAGs).

| Groups | Bin Id | Scaffolds Num | N50 (scaffolds) | Mean scaffold length | Completeness (%) | Contamination | Strain heterogeneity | Domain |
| --- | --- | --- | --- | --- | --- | --- | --- | --- |
| wild habitat environment group (CK group) | **G_**bin2 | 4729 | 2289 | 2331.125396 | 100 | 250.75 | 23.44 | Bacteria |
|  | **G_**bin3 | 15389 | 2167 | 2260.614205 | 100 | 435.33 | 4.06 | Bacteria |
|  | **G_**bin5 | 1336 | 2144 | 2179.508982 | 82.76 | 51.38 | 77.65 | Bacteria |
|  | **G_**bin8 | 1187 | 1933 | 1987.730413 | 58.04 | 19.25 | 13.21 | Bacteria |
|  | **G_**bin10 | 4686 | 1989 | 2087.925523 | 92.29 | 127.98 | 14.08 | Bacteria |
| agriculture group (WA group) | **G_**bin3 | 814 | 7520 | 5614.399263 | 99.14 | 75.94 | 73.17 | Bacteria |
|  | **G_**bin4 | 1195 | 4659 | 3890.933891 | 88.5 | 147.68 | 89.45 | Bacteria |
|  | **G_**bin5 | 1479 | 4702 | 4019.001352 | 87.66 | 37.18 | 87.74 | Bacteria |
|  | **G_**bin6 | 5095 | 3505 | 3195.286359 | 95.83 | 422.3 | 35.05 | Bacteria |
|  | **G_**bin16 | 463 | 6424 | 4833.779698 | 90.93 | 6.86 | 20.83 | Bacteria |
|  | **G_**bin21 | 5114 | 2843 | 2749.078021 | 98.68 | 250.21 | 26.62 | Bacteria |
|  | **G_**bin24 | 4241 | 2031 | 2092.649375 | 89.18 | 163 | 17.1 | Bacteria |
|  | **G_**bin30 | 10158 | 2438 | 2466.33737 | 100 | 540.18 | 13.03 | Bacteria |
|  | **G_**bin31 | 1683 | 4484 | 3708.845514 | 82.57 | 51.69 | 52.75 | Bacteria |
|  | **G_**bin33 | 2502 | 3442 | 3224.388489 | 92.87 | 156.38 | 70.47 | Bacteria |
|  | **G_**bin34 | 524 | 12186 | 7907.875954 | 93.86 | 55.37 | 81.03 | Bacteria |
|  | **G_**bin38 | 2271 | 2422 | 2418.09203 | 77.52 | 98.39 | 33.89 | Bacteria |
|  | **G_**bin39 | 957 | 2969 | 2863.823406 | 74.12 | 17.17 | 78.57 | Bacteria |
|  | **G_**bin40 | 687 | 4219 | 3676.762737 | 78.84 | 12.54 | 93.55 | Bacteria |
|  | **G_**bin46 | 1790 | 2654 | 2598.111732 | 84.56 | 42.18 | 34.83 | Bacteria |
|  | **G_**bin51 | 983 | 3499 | 3165.931841 | 85.34 | 61.6 | 97.65 | Bacteria |
| grazing group (WG group) | **G_**bin3 | 674 | 11298 | 7363.172107 | 90.43 | 85.38 | 67.5 | Bacteria |
|  | **G_**bin10 | 749 | 5757 | 4659.930574 | 72.41 | 3.45 | 50 | Bacteria |
|  | **G_**bin12 | 1861 | 2633 | 2569.73885 | 84.33 | 39.37 | 52.05 | Bacteria |
|  | **G_**bin14 | 600 | 3413 | 3212.978333 | 50.2 | 3.45 | 100 | Bacteria |
|  | **G_**bin20 | 1730 | 4396 | 3819.369364 | 85.19 | 70.34 | 64.36 | Bacteria |
|  | **G_**bin22 | 11147 | 2532 | 2562.108819 | 100 | 636.12 | 17.1 | Bacteria |
|  | **G_**bin31 | 7131 | 2635 | 2611.722059 | 94.83 | 335.55 | 34.5 | Bacteria |
|  | **G_**bin33 | 1087 | 5236 | 4339.825207 | 86.92 | 58.12 | 73.33 | Bacteria |
|  | **G_**bin39 | 1788 | 3509 | 3211.338367 | 83.26 | 126.78 | 59.12 | Bacteria |
|  | **G_**bin41 | 13927 | 3326 | 3187.221512 | 100 | 967.57 | 26.46 | Bacteria |
|  | **G_**bin44 | 1822 | 3195 | 3028.925357 | 91.39 | 111.8 | 47.24 | Bacteria |
|  | **G_**bin48 | 1233 | 2386 | 2365.885645 | 66.18 | 25 | 89.47 | Bacteria |
|  | **G_**bin50 | 535 | 8907 | 6512.614953 | 93.02 | 29.62 | 76.25 | Bacteria |
|  | **G_**bin54 | 1811 | 3638 | 3327.734401 | 97.41 | 66.07 | 86.75 | Bacteria |
|  | **G_**bin57 | 1132 | 3654 | 3317.855124 | 94.42 | 70.95 | 35.92 | Bacteria |
| domestic sewage group (WS group) | **G_**bin3 | 1008 | 3511 | 3183.513889 | 87.93 | 60.5 | 85.39 | Bacteria |
|  | **G_**bin4 | 1764 | 2198 | 2229.400227 | 70.69 | 23.16 | 48 | Bacteria |
|  | **G_**bin6 | 983 | 2382 | 2386.045778 | 56.15 | 22.47 | 84.62 | Bacteria |
|  | **G_**bin7 | 2706 | 2447 | 2433.620103 | 95.92 | 141.91 | 16.04 | Bacteria |
|  | **G_**bin9 | 4485 | 1984 | 2057.789521 | 93.1 | 254.41 | 13.46 | Bacteria |
|  | **G_**bin12 | 1316 | 3109 | 2971.75152 | 77.19 | 57.91 | 92.19 | Bacteria |
|  | **G_**bin14 | 1264 | 2191 | 2301.984968 | 50.09 | 16.93 | 11.43 | Bacteria |
|  | **G_**bin16 | 1562 | 2316 | 2357.412292 | 64.89 | 47.18 | 56.1 | Bacteria |
|  | **G_**bin20 | 1435 | 2203 | 2240.240418 | 69.04 | 30.74 | 51.9 | Bacteria |
|  | **G_**bin23 | 813 | 3666 | 3268.153752 | 82.43 | 19.38 | 81.4 | Bacteria |
|  | **G_**bin24 | 1664 | 2689 | 2648.963942 | 93.73 | 85.98 | 79.61 | Bacteria |
|  | **G_**bin25 | 951 | 2972 | 2872.900105 | 76.29 | 37.8 | 69.57 | Bacteria |
|  | **G_**bin27 | 384 | 19299 | 11756.58333 | 98.17 | 0.9 | 77.78 | Bacteria |


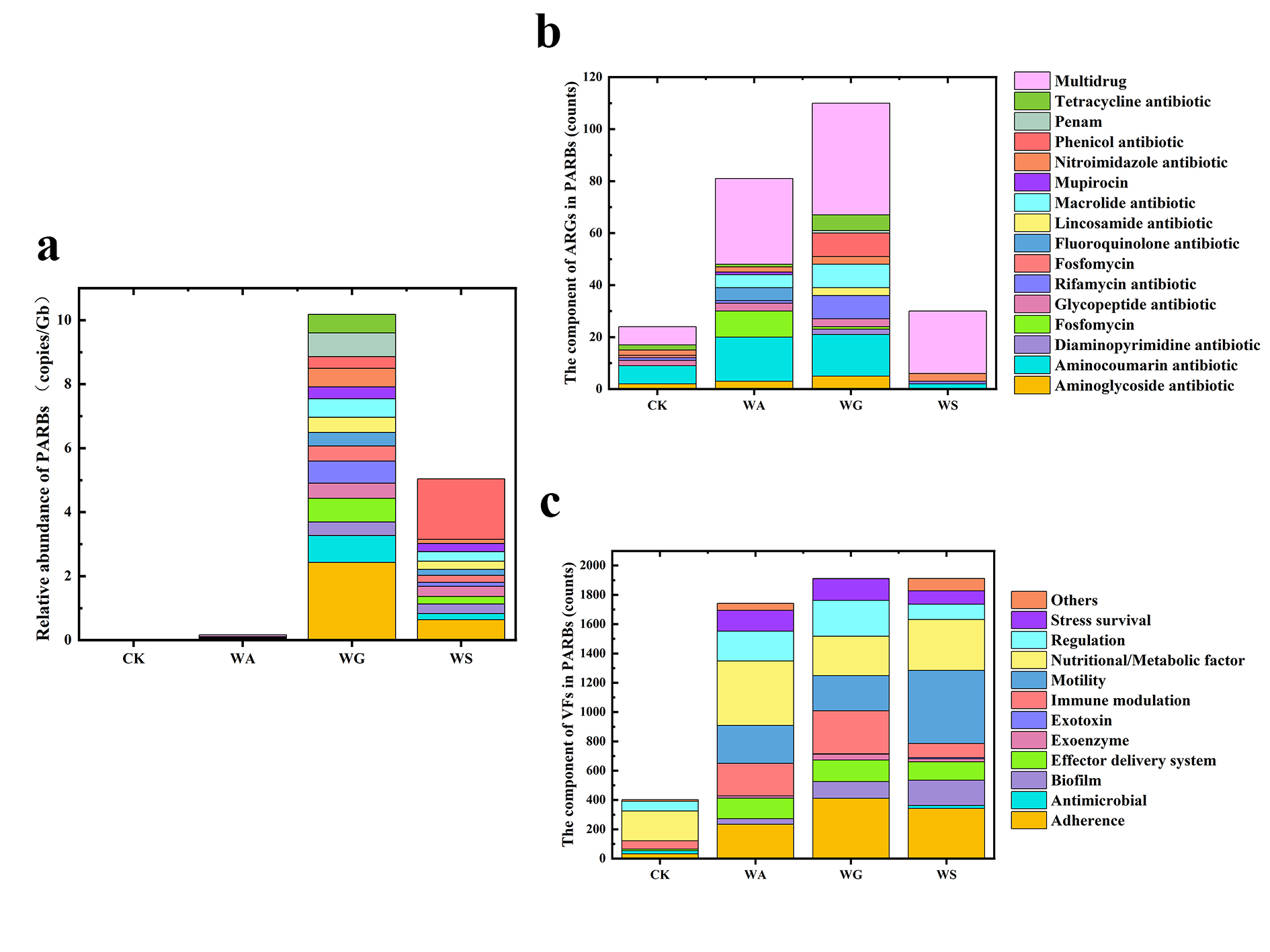


Figure S1 Characteristics of pathogenic antibiotic-resistant bacteria (PARBs).

Note: (a) The total abundance of PARBs. (b) The component and total numbers of antibiotic resistance genes (ARGs) in PARBs. (c) The component and total numbers of Virulence genes (VFs) in PARBs.

Table S2 Contributions of ARGs and VFs to PARBs.

| Groups | BIN ID | F1 | F2 | F3 | F4 | F5 | F6 | F7 | F8 | F9 | TFS | HR | THR |
| --- | --- | --- | --- | --- | --- | --- | --- | --- | --- | --- | --- | --- | --- |
| CK | **G_bin2** | 0.597 | 8.2 | -4.017 | -1.742 | -1.503 | -4.183 | 6.683 | -6.062 | 8.923 | 6.896 | 0.003 | 0.067 |
|  | **G_bin3** | 3.917 | -1.038 | -10.964 | 45.451 | -1.489 | 20.432 | 10.065 | -2.537 | 2.593 | 66.43 | 0.07 |  |
|  | **G_bin5** | -2.554 | -0.692 | -0.86 | 8.862 | -9.125 | -0.04 | 5.819 | -5.449 | -0.154 | -4.194 | 0 |  |
|  | **G_bin8** | -4.071 | -13.196 | -2.944 | -3.983 | -5.579 | 0.248 | 2.753 | -1.682 | -3.041 | -31.496 | -0.002 |  |
|  | **G_bin10** | 3.877 | -10.399 | -4.651 | -1.978 | -3.272 | -0.286 | 2.667 | -1.633 | 5.105 | -10.57 | -0.004 |  |
| WA | **G_bin3** | -4.084 | 6.018 | -18.647 | 12.478 | 17.554 | 0.516 | -19.352 | 2.05 | -3.651 | -7.118 | -0.068 | -1.55 |
|  | **G_bin4** | 6.578 | -2.434 | 8.163 | -4.452 | -4.56 | 1.486 | -1.823 | -0.076 | -7.352 | -4.469 | -0.024 |  |
|  | **G_bin5** | 2.169 | 5.423 | -12.008 | -3.637 | 0.589 | -5.955 | -6.754 | -1.759 | 1.828 | -20.105 | -0.324 |  |
|  | **G_bin6** | -5.806 | 22.134 | -5.51 | 14.01 | -10.06 | -30.556 | 3.076 | -2.654 | 3.266 | -12.1 | -0.072 |  |
|  | **G_bin16** | 1.916 | -6.459 | -3.271 | -5.484 | -1.952 | 7.218 | -2.032 | -1.263 | 4.703 | -6.624 | -0.091 |  |
|  | **G_bin21** | -5.254 | -5.959 | -0.609 | 0.198 | -2.937 | -2.08 | 0.186 | -3.119 | -2.853 | -22.427 | -0.094 |  |
|  | **G_bin24** | -2.468 | -7.736 | 7.614 | -2.154 | 0.895 | 5.907 | 3.289 | -6.071 | -7.143 | -7.867 | -0.056 |  |
|  | **G_bin30** | -3.29 | -3.757 | -8.008 | -6 | 7.898 | 10.004 | -14.458 | -10.537 | 16.519 | -11.628 | -0.083 |  |
|  | **G_bin31** | -8.08 | -14.017 | -12.727 | 7.338 | -5.004 | -5.66 | 4.911 | 26.253 | 1.022 | -5.963 | -0.018 |  |
|  | **G_bin33** | -1.381 | -12.528 | 1.406 | -0.252 | -1.673 | -0.907 | 0.515 | -1.32 | -0.949 | -17.089 | -0.081 |  |
|  | **G_bin34** | -0.875 | 9.545 | -7.449 | 2.316 | -0.138 | -8.263 | -12.596 | -2.72 | 1.961 | -18.218 | -0.13 |  |
|  | **G_bin38** | -0.828 | 2.925 | 10.125 | -5.266 | -1.691 | 2.67 | 0.049 | 0.888 | 0.582 | 9.455 | 0.068 |  |
|  | **G_bin39** | -5.347 | -10.571 | -6.332 | -3.211 | -3.736 | -0.581 | 1.881 | -0.04 | 2.353 | -25.584 | -0.168 |  |
|  | **G_bin40** | -4.7 | -6.275 | -6.722 | -1.84 | -2.187 | -0.873 | -3.596 | -3.535 | 3.748 | -25.98 | -0.356 |  |
|  | **G_bin46** | -5.147 | 3.066 | 5.378 | 2.282 | 2.843 | -4.857 | -4.167 | -8.751 | 0.736 | -8.617 | -0.036 |  |
|  | **G_bin51** | -8.121 | 1.508 | 2.097 | -4.445 | 1.512 | 0.48 | -2.352 | 0.276 | 8.742 | -0.304 | -0.015 |  |
| WG | **G_bin3** | 5.046 | 9.446 | -11.761 | 4.745 | 11.08 | -4.326 | -2.277 | 4.022 | -8.666 | 7.308 | 17.734 | 87.93 |
|  | **G_bin10** | -2.6 | 15.441 | 21.147 | -2.345 | -5.605 | 5.911 | -13.8 | 3.636 | -6.009 | 15.777 | 13.318 |  |
|  | **G_bin12** | -4.772 | 6.23 | 3.202 | 0.963 | -0.727 | -5.659 | 0.184 | -6.263 | -4.598 | -11.44 | -4.828 |  |
|  | **G_bin14** | -6.286 | -7.385 | 0.812 | -3.166 | -4.489 | 1.197 | 0.223 | 3.769 | 0.932 | -14.394 | -10.631 |  |
|  | **G_bin20** | 3.977 | 6.013 | -3.233 | -2.518 | -3.233 | -1.599 | -5.193 | 0.7 | -1.775 | -6.862 | -3.258 |  |
|  | **G_bin22** | 61.948 | 42.138 | -24.066 | -18.642 | -10.829 | 10.663 | 7.726 | 2.246 | -0.173 | 71.012 | 48.703 |  |
|  | **G_bin31** | 1.586 | -7.293 | -4.421 | -2.35 | -1.102 | 1.787 | -1.646 | -0.286 | -3.082 | -16.805 | -7.979 |  |
|  | **G_bin33** | -0.987 | 9.493 | -5.658 | 6.578 | 10.412 | -2.321 | 5.303 | -0.338 | -3.986 | 18.496 | 7.807 |  |
|  | **G_bin39** | -4.757 | -10.888 | -1.358 | -3.149 | -2.868 | 1.748 | 1.641 | -0.122 | -1.921 | -21.674 | -10.291 |  |
|  | **G_bin41** | 81.651 | -30.418 | 25.978 | 10.281 | 9.347 | -10.714 | -2.579 | 1.526 | 4.745 | 89.816 | 52.124 |  |
|  | **G_bin44** | 2.026 | 6.315 | -5.817 | 5.309 | 8.554 | 3.312 | -5.232 | 3.307 | -6.937 | 10.837 | 4.002 |  |
|  | **G_bin48** | -6.234 | -10.278 | -2.182 | -2.923 | -3.532 | -0.888 | 1.623 | -0.311 | -0.682 | -25.407 | -14.744 |  |
|  | **G_bin50** | 2.401 | -2.93 | -1.837 | -5.203 | -1.839 | 3.676 | -1.324 | 2.944 | -3.721 | -7.833 | -2.893 |  |
|  | **G_bin54** | -5.793 | 5.598 | 7.16 | -3.07 | 0.952 | -0.985 | -2.468 | 2.653 | -4.364 | -0.317 | -0.234 |  |
|  | **G_bin57** | 3.042 | -7.848 | -3.652 | -1.759 | 6.84 | 1.497 | 0.536 | 3.421 | -3.627 | -1.55 | -0.9 |  |
| WS | **G_bin3** | -9.725 | 20.109 | 18.868 | -8.224 | 14.536 | 3.993 | 1.82 | 8.916 | 2.104 | 52.397 | 33.439 | 153.53 |
|  | **G_bin4** | -5.931 | -13.262 | -5.763 | -4.459 | -3.216 | 0.656 | 1.697 | 0.845 | 2.158 | -27.275 | -5.222 |  |
|  | **G_bin6** | -7.749 | -8.431 | 1.608 | -5.543 | -0.252 | 0.856 | 2.62 | 0.931 | 1.91 | -14.051 | -4.185 |  |
|  | **G_bin7** | -6.102 | -9.311 | -4.566 | -5.128 | -0.572 | -0.196 | 4.856 | 0.745 | 2.118 | -18.155 | -4.248 |  |
|  | **G_bin9** | 1.772 | -0.338 | 10.717 | 3.074 | -6.523 | 1.72 | 1.394 | -13.028 | -14.892 | -16.106 | -5.139 |  |
|  | **G_bin12** | -5.889 | -11.346 | -4.258 | -2.759 | -3.289 | -0.792 | 2.442 | -0.318 | 0.2 | -26.008 | -3.32 |  |
|  | **G_bin14** | -9.667 | -0.391 | 7.115 | -5.093 | 3.324 | 0.109 | 4.499 | 1.555 | 3.968 | 5.418 | 1.153 |  |
|  | **G_bin16** | -2.35 | -7.07 | -4.403 | -2.871 | -5.536 | -1.345 | -0.741 | -1.02 | -4.074 | -29.409 | -5.631 |  |
|  | **G_bin20** | -6.47 | -1.091 | 3.679 | -4.647 | 0.479 | 1.289 | -0.411 | 3.262 | 1.329 | -2.581 | -0.659 |  |
|  | **G_bin23** | -7.626 | -2.895 | 4.95 | -3.406 | -2.559 | 1.278 | -2.082 | 4.743 | 1.298 | -6.297 | -1.875 |  |
|  | **G_bin24** | -9.053 | 4.313 | 18.803 | 2.031 | -3.669 | 6.132 | 1.433 | 3.911 | 2.589 | 26.49 | 6.762 |  |
|  | **G_bin25** | -9.398 | 8.726 | 1.626 | -8.817 | 25.266 | -3.936 | 24.94 | -5.296 | 0.07 | 33.181 | 4.235 |  |
|  | **G_bin27** | -9.106 | 33.594 | 27.247 | 14.601 | -7.335 | 2.217 | 0.052 | 3.588 | 8.147 | 73.005 | 138.22 |  |

Table S3 Rotating component matrix in factor analysis.

|  | ARGs or VFGs | F1 | F2 | F3 | F4 | F5 | F6 | F7 | F8 | F9 |
| --- | --- | --- | --- | --- | --- | --- | --- | --- | --- | --- |
| ARGs | Aminoglycoside antibiotic | 0.615 | 0.544 | 0.012 | 0.073 | -0.126 | -0.134 | -0.07 | 0.051 | 0.032 |
|  | Aminocoumarin antibiotic | 0.09 | 0.586 | -0.14 | -0.094 | 0.138 | -0.058 | 0.018 | -0.028 | 0.547 |
|  | Diaminopyrimidine antibiotic | 0.978 | -0.11 | -0.033 | -0.044 | -0.019 | -0.036 | -0.008 | 0.058 | -0.008 |
|  | Fosfomycin | -0.057 | -0.083 | -0.004 | 0.003 | -0.059 | 0.191 | -0.086 | -0.004 | 0.855 |
|  | Glycopeptide antibiotic | -0.017 | -0.01 | -0.17 | 0.016 | 0.598 | 0.699 | -0.136 | -0.158 | 0.043 |
|  | Rifamycin antibiotic | 0.067 | 0.932 | 0 | 0.1 | 0.074 | 0.107 | 0.047 | 0.04 | -0.055 |
|  | Fosfomycin | -0.002 | 0.045 | -0.133 | -0.024 | 0.89 | 0.064 | -0.047 | 0.053 | -0.008 |
|  | Fluoroquinolone antibiotic | 0.02 | -0.127 | -0.063 | 0.687 | -0.053 | -0.072 | -0.15 | -0.333 | -0.171 |
|  | Lincosamide antibiotic | 0.921 | 0.326 | 0 | -0.003 | -0.04 | -0.045 | -0.036 | 0.063 | -0.023 |
|  | Macrolide antibiotic | 0.921 | -0.134 | -0.067 | -0.02 | -0.027 | 0.018 | -0.016 | 0.086 | 0.041 |
|  | Mupirocin | 0.016 | -0.042 | -0.001 | -0.118 | 0.063 | 0.126 | -0.199 | -0.626 | -0.339 |
|  | Nitroimidazole antibiotic | 0.216 | 0.189 | 0.007 | -0.033 | -0.064 | -0.111 | -0.243 | 0.604 | -0.285 |
|  | Phenicol antibiotic | 0.579 | 0.496 | -0.063 | -0.019 | -0.096 | 0.24 | -0.069 | 0.05 | -0.101 |
|  | Penam | 0.087 | 0.942 | 0.066 | 0.081 | -0.051 | -0.027 | -0.062 | 0.024 | -0.034 |
|  | Tetracycline antibiotic | 0.922 | 0.129 | -0.074 | -0.08 | 0.146 | -0.026 | -0.046 | 0.085 | -0.05 |
|  | Multidrug | 0.274 | -0.067 | 0.187 | -0.198 | 0.22 | 0.135 | -0.049 | 0.703 | -0.086 |
| VFs | Adherence | 0.003 | 0.297 | 0.839 | -0.057 | -0.072 | 0.108 | 0.052 | 0.033 | -0.069 |
|  | Antimicrobial | -0.036 | -0.034 | 0.366 | 0.205 | 0.726 | -0.205 | -0.014 | 0.036 | -0.028 |
|  | Biofilm | -0.072 | -0.124 | 0.879 | 0.054 | 0.041 | -0.039 | -0.052 | -0.005 | -0.047 |
|  | Effector delivery system | -0.088 | -0.237 | 0.533 | 0.423 | -0.088 | 0.027 | 0.054 | 0.359 | -0.091 |
|  | Exoenzyme | -0.019 | 0.002 | -0.017 | 0 | -0.01 | 0.779 | 0.455 | 0.009 | 0.046 |
|  | Exotoxin | -0.053 | -0.002 | -0.044 | 0.098 | -0.069 | 0.18 | 0.828 | 0.02 | -0.194 |
|  | Immune modulation | 0.038 | 0.505 | 0.261 | 0.544 | 0.038 | 0.176 | -0.316 | 0.052 | 0.1 |
|  | Motility | -0.058 | 0.016 | 0.784 | 0.116 | 0.064 | -0.025 | 0.207 | 0.066 | 0.085 |
|  | Nutritional/Metabolic factor | -0.091 | 0.055 | 0.134 | 0.886 | 0.244 | 0.039 | 0.161 | 0.039 | 0.101 |
|  | Regulation | -0.05 | 0.427 | 0.067 | 0.736 | -0.055 | 0.208 | 0.122 | 0.108 | 0.013 |
|  | Stress survival | -0.042 | 0.182 | 0.206 | 0.288 | -0.253 | 0.657 | -0.106 | -0.013 | 0.254 |
|  | Others | -0.067 | -0.066 | 0.344 | -0.062 | -0.028 | -0.064 | 0.774 | -0.035 | 0.14 |
| Variance Contributions (%) | | 15.72 | 12.497 | 10.311 | 9.021 | 7.026 | 6.729 | 6.659 | 5.616 | 5.185 |

Note: The F1, F2, F3, F4, F5, F6, F7, F8 and F9 are the factor loading of factor 1, factor 2, factor 3, factor 4, factor 5, factor 6, factor 7, factor 8 and factor 9.

Table S4 Content of environmental factors.

|  | TN (g/kg) | RC (g/kg) | TK (g/kg) | TP  (g/kg) | SON (mg/kg) | AP (mg/kg) | AK (mg/kg) | SAK (mg/kg) | pH |
| --- | --- | --- | --- | --- | --- | --- | --- | --- | --- |
| CK | 0.476 | 7.545 | 17.865 | 0.604 | 68.796 | 9.233 | 97.667 | 869.333 | 8.01 |
| WA | 1.892 | 76.389 | 18.275 | 1.510 | 205.065 | 89.1 | 468 | 944 | 8.187 |
| WG | 3.566 | 48.255 | 19.958 | 1.334 | 196.245 | 70.2 | 199.333 | 1005.333 | 8.197 |
| WS | 4.795 | 46.023 | 21.491 | 1.060 | 161.7 | 38.633 | 111.333 | 608.889 | 8.007 |
